# The fast and the studious? Ramadan observance and student performance

**DOI:** 10.1007/s00148-026-01204-9

**Published:** 2026-10-06

**Authors:** Kyra Hanemaaijer, Olivier Marie, Marco Musumeci

**Affiliations:** 1 University of Gothenburg, Gothenburg, Sweden; 2 Erasmus University Rotterdam & TI, CEPR, IZA, and CESifo, Rotterdam, Netherlands; 3 University of Padova, Padua, Italy

**Keywords:** Education, Religion, Fasting, Ramadan, Minority students, The Netherlands, I21, I24, Z12, J15

## Abstract

We examine how religious observance affects educational performance by studying the years in which Ramadan overlapped with the national end-of-secondary-school exams in the Netherlands. Using administrative microdata and a difference-in-differences design, we find that students who are most likely to fast obtain lower exam scores and are more likely to fail to graduate. As expected given the structure of the Dutch exam system, the increase in failure risk is concentrated among students near the pass threshold. Effects are larger in more religiously segregated schools. For longer-term outcomes, we document a substantially higher likelihood of dropping out among students aged 18 and older, who are legally permitted to leave school and therefore at risk of doing so. Finally, analyses of exam timing suggest that modest adjustments—such as avoiding scheduling two exams on the same day during Ramadan—may help reduce the disadvantages experienced by observant students.

**Supplementary Information:**

The online version contains supplementary material available at https://doi.org/10.1007/s00148-026-01204-9.

Do you think there are possible effects of taking tests and exams during periods of religious fasting? If so, do these effects form a possible obstacle to the progress of pupils and/or students in education?

*Tunahan Kuzu, Member of the Dutch Parliament*

It is the individual responsibility of a student to prepare as well as possible for tests and exams. It may be that students find religious obligations important, even during periods when tests are held. I leave that decision to the individual.

*Sander Dekker, Secretary of State at the Dutch Ministry of Education.*Question time at the Dutch Parliament on the $6^{th}$ of June 2016

## Introduction

Religious and civic obligations do not always align. This tension is especially salient for religious minorities, including the quarter of the world’s Muslims who live outside Muslim-majority countries (Lugo et al., 2011). During the ninth month of the Islamic calendar, Ramadan, healthy post-pubertal Muslims refrain from eating and drinking between sunrise and sunset for up to 30 days. In Muslim-minority contexts, public institutions rarely adjust to this rhythm. While adults may shift working hours or take leave, students sitting nationally scheduled exams cannot. As a consequence, Muslim students—who are already part of an under-privileged group—are required to take high-stakes exams while observing Ramadan. The potential repercussions on their academic performance have drawn media attention across Western countries.^1^

This paper provides the first large-scale causal evidence on the effect of Ramadan fasting on students’ exam performance in a Western, non-Muslim-majority context where no institutional accommodation is made for religious observance. We study the Netherlands, where about 5% of the population—mostly of Moroccan and Turkish descent—identifies as Muslim. National secondary-school graduation exams, which determine access to tertiary education, are held each May on fixed dates. In 2018 and 2019, for the first time in decades, these exams coincided with Ramadan. Earlier and later cohorts took identical exams outside the fasting period. Because both exam timing and Ramadan dates are determined exogenously, the overlap affected entire cohorts only in those 2 years, generating a sharp natural experiment on how religious observance interacts with educational productivity.

Prima facie, fasting might reduce concentration and learning ability. Laboratory studies link short-term fasting and dehydration to cognitive impairment and show that Ramadan observance alters sleep duration and quality, though small experimental studies offer mixed evidence.^2^ Whether these physiological disruptions translate into measurable educational outcomes is ultimately an empirical question—one that can rarely be answered convincingly because fasting behaviour and exam timing almost never vary independently. In our setting, however, they do.

We exploit this coincidence using a difference-in-differences (DID) framework comparing changes in outcomes for students more likely to fast with those for non-Muslim students across years when Ramadan did or did not overlap with exams. The roughly 11-day annual shift of the lunar calendar creates quasi-random timing variation, uncorrelated with ability, schools, or local trends. Parallel pre-trends are verified visually and with “HonestDiD” confidence bands, confirming the identifying assumption.

Because fasting cannot be directly observed, we construct a measure of fasting likelihood using a transparent machine learning (ML) model trained on Dutch survey data on religiosity among Moroccan- and Turkish-origin individuals. The model predicts fasting probabilities based on demographic and neighbourhood characteristics such as mosque proximity, income, and ethnic composition. This measure captures meaningful within-group heterogeneity while avoiding coarse classifications based solely on nationality or name. Estimates are nearly identical when treatment is defined by Moroccan background alone, demonstrating that our findings reflect genuine behavioural variation rather than modelling choices.

Using administrative microdata on nearly 800,000 students taking national exams between 2014 and 2019, we find that Ramadan exposure significantly reduces academic achievement. Grades of students who are most likely to fast fall by 0.058 standard deviations, and their probability of failing to graduate increases by about 8%. These effects are robust to rich controls for prior ability, family background, and neighbourhood characteristics. A simpler DID using Moroccan-background status produces almost identical estimates.

The consequences are largest for lower-achieving students and those in more segregated schools, where fasting peers may amplify performance declines. In the most segregated quartile, grades fall by 7% of a standard deviation, and failure rates increase by 12%. We also observe longer-term effects: among students aged 18 or older—those legally allowed to leave school—the share dropping out the following year rises by about 22% relative to baseline, equivalent to an increase of roughly 1.7 percentage points on a baseline dropout rate of around 7.6%. Younger students under compulsory schooling show no comparable effect.

A range of checks reinforces the causal interpretation. There are no differences in baseline ability or continuous school-exam performance, and results are unchanged by alternative clustering choices, family fixed effects, or placebo tests among non-Muslim students. Mechanism analyses point to cognitive fatigue as the dominant channel. Students must wake before dawn and often sleep after the late-night meal, reducing sleep quality and duration. Consistent with this, afternoon exams are less affected by Ramadan than morning exams when taken in isolation, but this advantage is eliminated when students also sit a morning exam on the same day. Losses are also larger for subjects relying on short-term recall rather than accumulated knowledge.

Our results suggest that institutional schedules can inadvertently disadvantage specific groups. Modest adjustments—such as preferring afternoon slots for individual exams during Ramadan and avoiding scheduling a morning exam on the same day as an afternoon exam—could mitigate these effects without granting group-specific privileges. More broadly, the findings highlight how differences in temporal constraints can shape measured productivity in education.

This study contributes to several strands of research. A growing literature examines the economic and social effects of religious observance. At the macro level, Campante and Yanagizawa-Drott (2015) and Montero and Yang (2022) document declines in short-run productivity during major religious holidays. At the individual level, Almond and Mazumder (2011) show that prenatal exposure to Ramadan fasting in the United States has adverse long-run health effects; van Ewijk (2011) document similar later-life consequences for those prenatally exposed in Indonesia; and Majid (2015) finds persistent reductions in labour-market and cognitive outcomes for the same Indonesian cohorts. Aydilek and Karaoğlan (2024) similarly find that men prenatally exposed to Ramadan in Turkey complete significantly less schooling, with effects concentrated among those exposed during summer Ramadans. Evidence on the contemporaneous effect of students’ own fasting on their education remains limited and mixed. Oosterbeek and van der Klaauw (2013) show negative effects for Dutch university students when Ramadan overlaps with course preparation before exams are taken. Hornung et al. (2023) instead exploit variation in prescribed fasting hours and find positive effects on PISA and TIMSS scores, but those tests occur after Ramadan has ended, are low-stakes, and are taken mostly in Muslim-majority environments. Their treatment proxy is the school-level share of Muslim-origin students, offering no link to actual fasting behaviour, and they do not examine pre-trends. These design features make it difficult to separate social-capital formation from the contemporaneous cognitive impacts of fasting. Finally, Nuryakin et al. (2022) study Indonesian university exams on dates partially accommodating Ramadan, again without formal validation of parallel trends and in a very different institutional context.

We make four contributions to this growing literature. First, we provide the first causal evidence on the contemporaneous effect of Ramadan fasting during high-stakes national exams in a Western, non-Muslim-majority context with no institutional accommodation. Second, we show that rigid institutional calendars can widen pre-existing achievement gaps for an already disadvantaged minority group, linking religious observance to the broader literature on educational inequality (Blanden et al., 2022). Third, we improve upon earlier proxies for Muslim identity by estimating individual fasting propensity from survey-based religiosity linked to administrative microdata using a transparent machine-learning model. This allows us to capture behavioural heterogeneity within Muslim-origin groups and to conduct unusually rich validation checks, including pre-trend assessments, sibling fixed-effects designs, and peer-composition spillover analyses. Fourth, we provide exploratory evidence on mechanisms based on variation in exam timing within fasting days, which suggests simple scheduling adjustments that could mitigate harm without introducing group-specific privileges.

The rest of the paper proceeds as follows. Section 2 provides background on Ramadan, Muslims in the Netherlands, the Dutch secondary-school system, and the overlap between exams and Ramadan. Section 3 describes the administrative data. Section 4 outlines the identification of the treated group, our difference-in-differences strategy, and the machine-learning model used to predict fasting compliance. Section 5 presents the main results, and Sect. 6 examines heterogeneity by student, school, and subject. Section 7 explores mechanisms and policy implications. Section 8 concludes.

## Background

### Ramadan

Ramadan is the ninth month of the Islamic lunar calendar and requires healthy Muslims who have reached puberty to abstain from eating and drinking between sunrise and sunset. Widely accepted exemptions—reflected in religious guidance and practice—apply in cases such as illness, pregnancy, menstruation, younger children, or travel. Because the lunar calendar is approximately 11 days shorter than the Gregorian calendar, Ramadan shifts earlier each year, causing substantial variation in daylight hours—and thus fasting duration—across years. In the Netherlands, the fast may last around 8 h in winter but up to 16–17 h in late spring or early summer.

In many Muslim-majority countries, institutions adjust working hours or school schedules during Ramadan. Such accommodations are largely absent in Western Europe, where most schools operate according to fixed national timetables. As Muslims currently constitute around 5% of the European population and are projected to reach 11% by 2050 (Lugo et al., 2011), the question of how schooling systems should respond to Ramadan observance has become increasingly salient.

### Muslims in the Netherlands

Around 5% of the Dutch population aged 15 or older identifies as Muslim. The two largest Muslim-origin communities are those with Turkish and Moroccan migration backgrounds, each representing roughly 2.5–2.6% of the Dutch population. These groups are strongly urbanized: about 45% of individuals with a Moroccan background and 35% of those with a Turkish background live in one of the four largest cities (Amsterdam, Rotterdam, The Hague, Utrecht), as illustrated in Appendix Fig. A1.

Because these communities are demographically younger than the native Dutch population, their representation among high-school students is substantially higher than in the population at large. In 2017, students with a Moroccan or Turkish migration background jointly accounted for 7.1% of all secondary-school exam takers (3.6% Moroccan; 3.5% Turkish).^3^ Other Muslim-origin groups—such as those from Afghanistan, Syria, Iraq, Somalia, or Iran—represent much smaller shares of the relevant cohorts, both because of their smaller overall population size and because many arrived only recently. As a result, they contribute only marginally to the composition of the exam-taking population in our data.^4^

### Secondary education in the Netherlands

Pupils in the Netherlands typically start secondary school at age 12 (Appendix Fig. A2 provides a graphical overview of the full Dutch education system). At the end of primary school, students take a standardized assessment—most commonly the CITO test during our study period—which, together with the teacher’s recommendation, informs the assignment into one of three tracks: pre-vocational secondary education (VMBO), senior general secondary education (HAVO), or pre-university education (VWO).^5^ The three tracks last 4, 5, and 6 years, respectively.

During the second or third year (depending on track), students choose a subject profile (*profiel*), which shapes the set of subjects they take in the remainder of secondary school. Some subjects, such as Dutch, English, and physical education, are compulsory across all profiles. Students are required to attend school until age 16, after which the requirement becomes conditional: they must remain in education until age 18 unless they obtain a so-called start qualification (a diploma from HAVO, VWO, or secondary vocational education).

To graduate, students must pass the national secondary school examination. They normally sit the exam between ages 16 and 18, depending on their track. The final grade consists of 50% continuous school-based assessments and 50% centralized national exams in May of the final year. Continuous assessments consist of assignments and tests administered over the previous 2 to 3 years and graded internally by teachers. In contrast, the centralized exams are identical nationwide and are graded by both the student’s own teacher and a randomly assigned teacher from another school. Students typically take between five and eight central exams, depending on their track and profile. Grades range from 1 to 10, with 5.5 as the minimum passing average in most cases, alongside additional track-specific graduation requirements.

### Central exams and Ramadan overlap

Central exams are administered each May over a 3-week period, following a national schedule set by the Ministry of Education (see *Examenblad.nl* for detailed schedules and regulations). A first retake session takes place in June: students who were seriously ill or faced other compelling personal circumstances may sit missed exams, and all students may resit one exam to improve their grade, with the highest grade counting. A second, smaller retake session is held in July for students who could not participate earlier.

In recent years, this May exam period has begun to overlap with Ramadan, as shown in Appendix Fig. A3. Until 2017, Ramadan always fell after the central exams and therefore did not coincide with either the exams themselves or the immediate preparation period.^6^ In 2018 and 2019, however, Ramadan overlapped directly with the central exams. The overlap was partial in 2018 since only the first three exam days preceded Ramadan, and complete in 2019.

During the overlapping years, fasting entailed long periods without food or drink. While the practical overlap with the exam timetable is determined by sunrise and sunset—around 05:50 and 21:30 in May—many observant Muslims begin fasting earlier at the *fajr* prayer. This implies very long fasting hours, often 16 h or more per day. Morning exams began at 09:00 and afternoon exams at 13:30, meaning that on days with a morning exam students had very limited sleep opportunity after the pre-dawn meal, and began afternoon exams after 7–10 h without food or drink.

Debate about the scheduling conflict predates our study period. In 2016, a Member of Parliament from the party DENK formally requested that the government reschedule central exams to avoid Ramadan. The State Secretary for Education rejected the proposal, stating that “it is practically impossible to reschedule the final exams to accommodate Ramadan” and emphasizing that preparation for exams is the responsibility of individual students.^7^

## Data

### Administrative data

We use linked administrative microdata from multiple registries compiled by Statistics Netherlands (CBS). Appendix C provides full details on data construction, sample definitions, and variable coding. Our analysis includes all secondary-school students who took the central final exam between 2014 and 2019 and for whom we observe the required variables. The main source of sample attrition is the national end-of-primary-school test (*CITO*), which we use as a standardized measure of ability and can be linked for roughly three-quarters of exam takers. The resulting dataset contains 841,376 exam sittings (789,259 unique students) from 549 schools; around 6.5% of students appear more than once because they repeated the final year.

For each student, we observe central exam grades (including and excluding retakes), continuous school-exam grades (overall and by subject), pass/fail status, track and subtrack choices, any grade repetition or track mobility, their CITO score, and demographic characteristics including birth date and migration background. These data are further enriched with linked information on parental income, employment, and health-related disability, as well as neighbourhood characteristics such as ethnic composition, welfare dependency, household size, fertility, and age structure.

Migration background follows the standard CBS definition: (i) first-generation immigrants are born abroad and have at least one parent born abroad; (ii) second-generation immigrants are born in the Netherlands with at least one parent born abroad; (iii) third-generation immigrants are born in the Netherlands to Dutch-born parents but have at least one grandparent born abroad.^8^ Everyone else is considered to have no migration background.

Appendix Fig. A4 shows the distribution of standardized final-exam grades in 2017 for Moroccan-background, Turkish-background, and other students. Even before Ramadan ever overlapped with the exams, Moroccan- and Turkish-background students scored about 0.48 and 0.50 standard deviations lower than students without a Muslim-majority background. Additional descriptives show that these students live in poorer and more segregated neighbourhoods, attend schools with higher concentrations of similar-background peers, and have significantly lower CITO scores. Together, these patterns underscore the structural disadvantage from which these groups enter secondary school and approach the high-stakes exam.

### Other data sources

To measure fasting behaviour and local religious context, we draw on two complementary data sources. First, we use the SIM Survey (*Survey Integratie Minderheden*) from 2006, 2011, 2015, and 2020, which contains detailed questions on religiosity and Ramadan observance. Because respondents can be linked to CBS administrative registers, we observe their demographic and socioeconomic characteristics in the same framework as our main dataset. After linkage and standard cleaning (Appendix C.4), the survey provides 5400 usable observations for individuals with a Moroccan or Turkish migration background; summary statistics appear in Appendix Table B2.

Second, we construct a dataset on mosque locations and characteristics by scraping geocoded information from moskeewijzer.nl (collected in 2021). This allows us to measure the distance between a student’s home neighbourhood and the nearest mosque, offering an additional spatial proxy for local religious infrastructure that we use to validate the fasting-probability model.

## Identification strategy

### Identification of the “treated” group

To causally identify the effect of Ramadan on educational outcomes, we want to compare students who are likely to fast during their final exams with students who are not. Although we do not observe fasting directly, we draw on detailed survey information on self-reported religiosity from 2670 Moroccan-origin and 2730 Turkish-origin individuals in the Netherlands. We focus on these two groups for two reasons. First, they are by far the largest Muslim-origin groups in the country: together they account for more than 7% of all high school exam takers in 2017, whereas the next largest Muslim-majority-origin groups are much smaller (e.g. Iraqi 0.6%, Afghan 0.5%, Syrian 0.2% of exam takers). Second, they are the only groups for which reliable survey-based information on Ramadan compliance exists. Other origin groups from predominantly Muslim countries are both numerically small and display substantial heterogeneity in religious practice. The SCP “Muslims in the Netherlands” surveys document, for example, that several such groups include sizeable secular minorities or non-Muslim subgroups despite originating from Muslim-majority contexts (Huijnk, 2019). Using country of origin alone would therefore be an unreliable proxy for actual fasting behaviour, motivating our more nuanced approach.

**Fig. 1:**
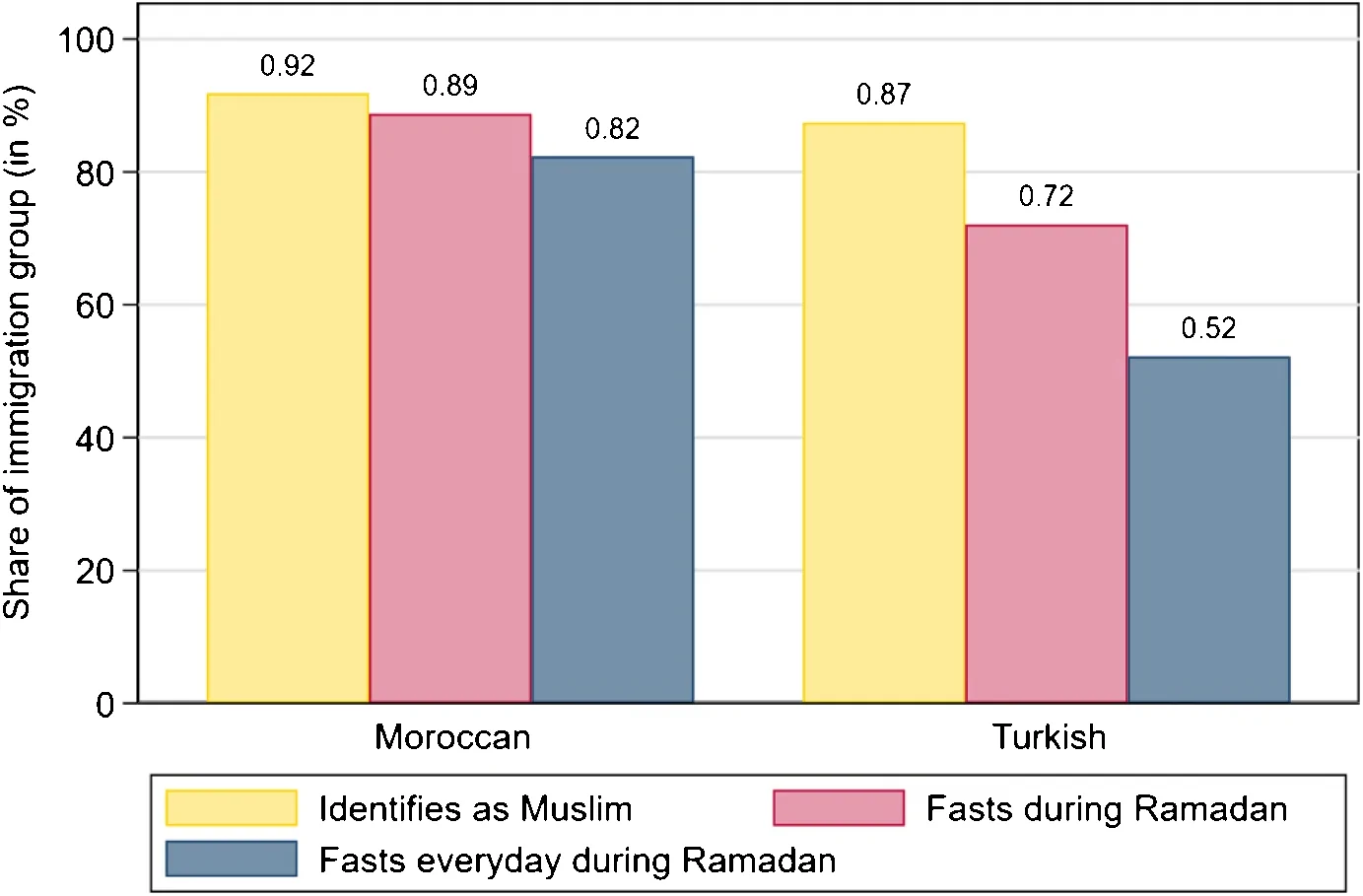
Religious identity and Ramadan fasting intensity by migration background. Note: Share of respondents who self-identify as Muslim (yellow), fast during Ramadan (red), and fast every day during Ramadan (blue), shown separately for those with a Moroccan (left) or Turkish (right) migration background. The sample comprises 2670 respondents with a Moroccan migration background and 2730 respondents with a Turkish migration background. Figures are based on SIM survey waves 2006, 2011, 2015, and 2020 linked to CBS microdata

Figure 1 documents that individuals with a Moroccan or Turkish migration background are very likely to identify as Muslim, but differ markedly in the strictness of fasting observance: 82% of Moroccan-background respondents report fasting every day during Ramadan, compared with 52% among those with a Turkish background. Similar patterns emerge across other indicators of Islamic practice (Appendix Table B1), consistently showing higher religious compliance among the Moroccan-origin population in the Netherlands. The SIM respondents closely resemble our sample of exam takers in observable characteristics (Appendix Table B2), with the exception that the SIM sample is older. Importantly, Appendix Fig. A5 shows that strict fasting behaviour is highly stable over the life course, including among 16–18-year-olds. This evidence alleviates concerns that our treatment assignment is driven by age-specific or survey-specific patterns rather than durable differences in Ramadan observance.

Individuals with a Moroccan migration background exhibit very high rates of full fasting compliance—over three-quarters in the SIM data—making them a clear component of the treated group. In contrast, fasting behaviour among those with a Turkish migration background is much more heterogeneous: only about half fast every day, while the rest fast irregularly or not at all. A classification rule based solely on country of origin would therefore misclassify a large share of Turkish-origin students, and would treat substantial secular minorities in both groups as fully treated. To overcome this limitation, we use the SIM survey to train a machine learning model that predicts each student’s probability of fasting every day during Ramadan based on rich individual and neighbourhood characteristics. We then assign these predicted compliance probabilities to all Moroccan- and Turkish-origin exam takers in our administrative data (details in Sect. 4.3).

Our main specification defines treatment as having a predicted probability above a high-compliance threshold, but we also estimate two alternative approaches: (i) using the continuous probability as a treatment-intensity measure and (ii) treating all Moroccan-origin students as the only treated group while excluding Turkish-origin students. In every case, the control group consists of students without a migration background from a Muslim-majority country (see Appendix C). Across these three definitions, the estimated Ramadan effects are highly consistent, providing strong reassurance that our findings are not an artefact of how the treated group is constructed and that the machine-learning approach adds precision without driving the results.

### Main econometric specification

To estimate how Ramadan fasting affects performance in the high-stakes graduation exams, we exploit the quasi-experimental variation arising from the gradual calendar shift of Ramadan described in Sect. 2.4. Treated and control students attend the same schools and sit the same exams at the same moment—the only source of differential exposure is whether Ramadan happens to coincide with their exam year. We therefore adopt a difference-in-differences (DiD) design comparing high-compliance students to students without a migration background from a Muslim-majority country, before and after Ramadan overlaps with the central exam. Our baseline specification is given in Eq. 1:

1 $$\begin{aligned} y_{i,t,s,p} = \alpha _{s,p}&+ \sum _{t=2014}^{2019} \beta _{t} \left( \textit{Treated}_i \times \textbf{1}\{ \text {ExamYear}=t \} \right) \nonumber \\&+ \zeta \,\textit{Treated}_i + \delta X_{i,t} + \gamma _t + \epsilon _{i,t,s,p} \end{aligned}$$

Here, $y_{i,t,s,p}$ is either the standardized central-exam grade (standardized within track × exam year) or a graduation indicator for student *i* in exam year *t*, school *s*, and subtrack *p*. $\textit{Treated}_i$ equals 1 for students with a high predicted probability of fasting in our preferred specification; in robustness analyses, it is instead (i) the continuous fasting-probability score or (ii) a dummy for Moroccan background only. $\textbf{1}\{\text {ExamYear}=t\}$ denotes a year dummy. The interactions $\textit{Treated}_i \times \textbf{1}\{\text {ExamYear}=t\}$ generate a coefficient $\beta _t$ for each exam year 2014–2019. These event-study coefficients allow us to test for parallel trends in the pre-Ramadan-overlap years (2014–2017) and to estimate the causal impact once Ramadan coincides with the exams (2018–2019). We therefore interpret $\beta _{2018}$ and $\beta _{2019}$ as the treatment effects.

We include school-track-subtrack fixed effects $\alpha _{s,p}$, exam-year fixed effects $\gamma _t$, and a rich set of individual, parental, and neighbourhood controls $X_{i,t}$: gender; age at exam; standardized ability (CITO score); standardized continuous-exam performance (omitted where used as a placebo outcome); prior grade-retention or upward track mobility; number of siblings; indicators for Moroccan, Turkish, and other Muslim-majority origins (dropped when used to define treatment); time spent abroad; parental income, unemployment, or illness benefits; and neighbourhood characteristics (share on social assistance, average household size, share below age 45, fertility rate, and shares of Moroccan and Turkish background residents). Standard errors are clustered at the school-track-subtrack-year level, our most conservative feasible choice given the institutional setting.^9^

Because Appendix Fig. A4 shows sizeable baseline gaps between treated and control groups, we verify that there are no differential compositional trends in observables when Ramadan overlaps with exams. Appendix Fig. A6 confirms stable trends in parental income, parental unemployment, and neighbourhood segregation. We also detect a small but positive secular improvement in ability among treated students—consistent with second-generation integration patterns—which would bias results in the opposite direction of our findings if left uncontrolled. This motivates including ability in all specifications.

For interpretability, we also estimate a more parsimonious before–after model:

2 $$\begin{aligned} y_{i,p,s,t} = \alpha _{p,s} + \beta \textit{Treated}_i \times \textit{Ramadan Year}_t + \zeta \textit{Treated}_i + \delta X_{i,t} +\gamma _t + \epsilon _{i, p, s, t} \end{aligned}$$

where $\textit{Ramadan Year}_t = 1$ in 2018–2019 only, and all other components and clustering match Eq. 1. Both specifications yield highly consistent estimates, no pre-trends, and strong statistical precision.

### Machine learning (ML) prediction of Ramadan compliance

A key empirical challenge is that we do not directly observe whether students fast during their final exams. To identify those most likely to comply with Ramadan obligations, we use the SIM Survey, which asks Moroccan- and Turkish-origin respondents in the Netherlands whether they fast every day during Ramadan.^10^ We train two separate prediction models—one for Moroccan-origin respondents and one for Turkish-origin respondents—reflecting their distinct fasting patterns documented in Sect. 4.1. This avoids imposing homogeneity across groups with different religious practices.

The models use a rich set of individual and neighbourhood characteristics, including age, gender, parental socioeconomic status, neighbourhood composition (shares of Moroccan and Turkish residents), and proximity to the nearest mosque. We estimate two common supervised-learning classifiers suited to moderately sized samples: penalized logistic regression and random forest.^11^ We evaluate predictive performance using a hold-out test sample (30% of SIM respondents). Penalized logistic regression consistently yields superior out-of-sample accuracy, and we therefore adopt it as our preferred model.

**Fig. 2:**
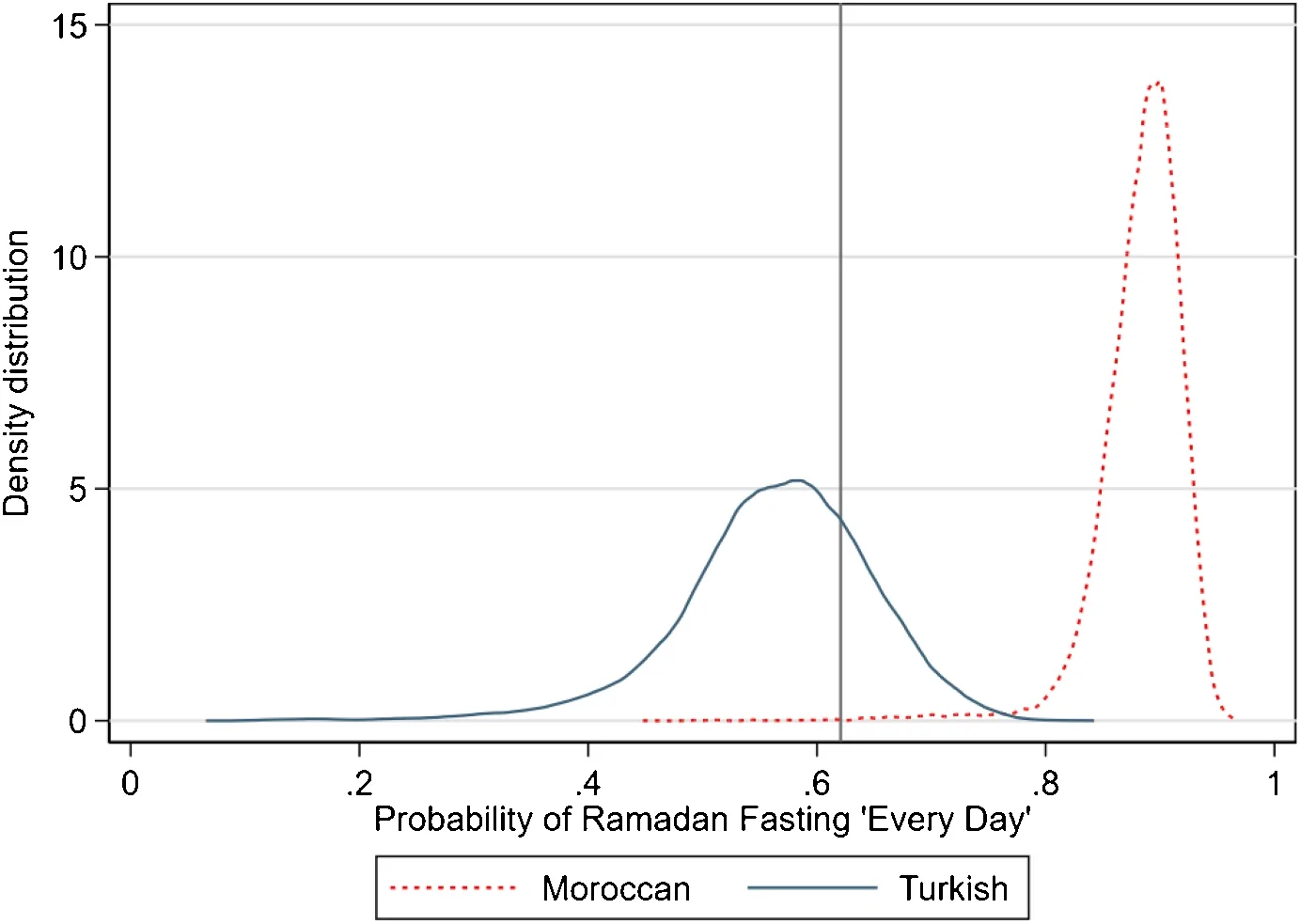
Predicted Ramadan compliance probabilities for exam takers. Notes: Density of predicted probability of fasting every day during Ramadan, estimated using penalized logistic regression trained on SIM survey data. Shown separately for Moroccan (red dashed) and Turkish (blue solid) migration backgrounds. The vertical line indicates the high-compliance threshold used in the main analysis

We apply the trained models to all Moroccan- and Turkish-origin exam takers in the CBS administrative data to generate student-specific probabilities of fasting every day during Ramadan. Students with no migration background from a Muslim-majority country are assigned a compliance probability of zero. Figure 2 shows the resulting distributions: probabilities for Moroccan-origin students are tightly clustered near one, whereas Turkish-origin students exhibit much greater heterogeneity. This aligns with validated survey evidence on compliance behaviour and reinforces the relevance of a continuous measure.

For interpretation and comparability, our preferred specification defines a treated student as one with a predicted probability above 0.62, the top quartile among Turkish-origin students (who have lower average compliance than Moroccan-origin students). Robustness checks demonstrate that results are highly stable when (i) varying the threshold or (ii) using the compliance probability as a continuous treatment-intensity measure.

We exclude exam takers from other Muslim-majority countries (e.g. Afghanistan, Iraq, Syria, Somalia) because we lack reliable information on fasting behaviour for these groups. Including them as treated would introduce substantial misclassification; assigning them to the control group would risk contamination. Appendix Table B3 documents the resulting composition of treatment across alternative specifications.

Overall, this machine learning approach ensures that treatment assignment reflects meaningful variation in religious practice, rather than imposing coarse assumptions based solely on country of origin.

## The impact of Ramadan on student performance

### Main results

**Fig. 3:**
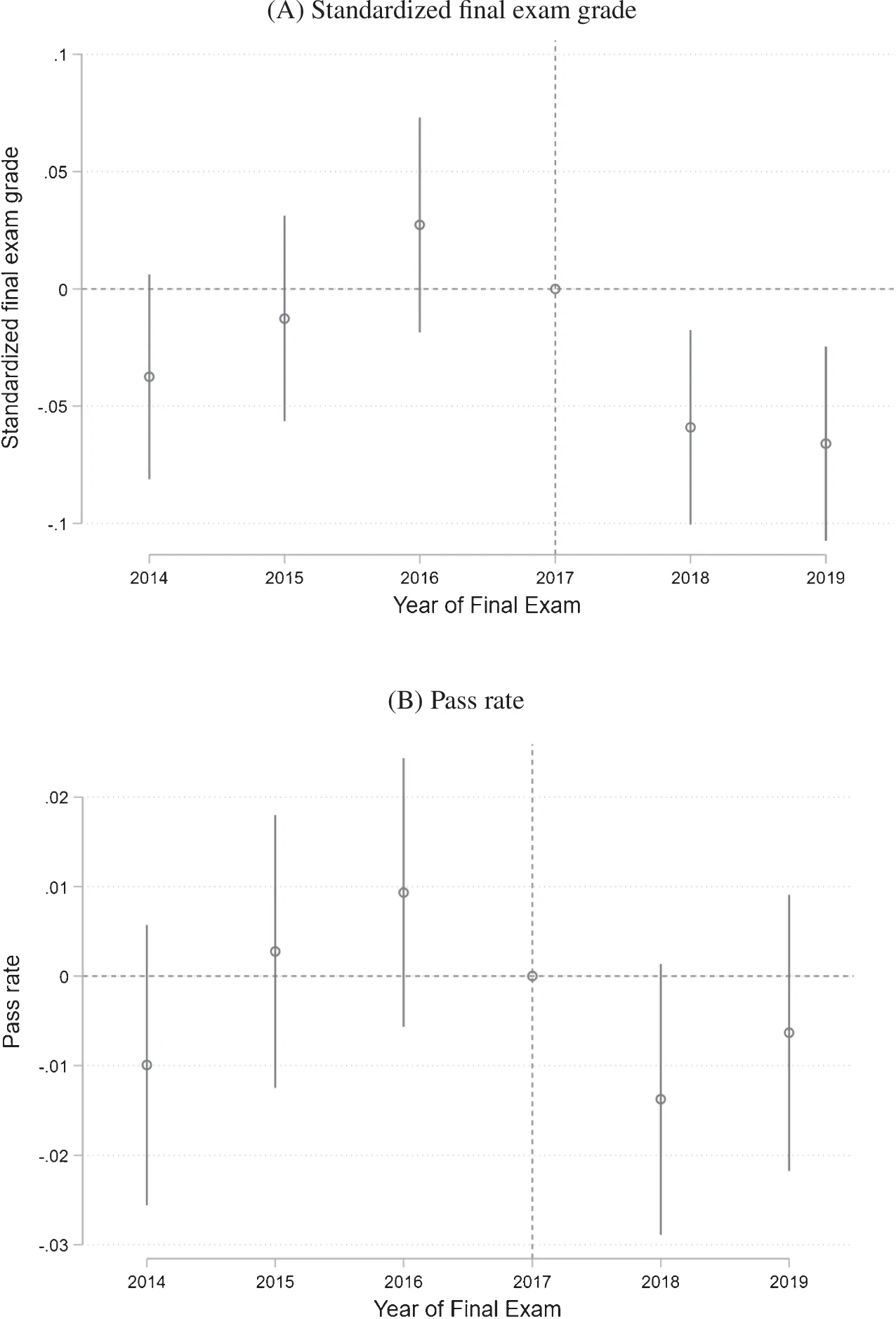
Impact of Ramadan on secondary education outcomes of students with a high Ramadan compliance probability. **A** Standardized final exam grade. **B** Pass rate. Notes: The panels plot the event–study coefficients $\beta _t$ and 99% confidence intervals from Eq. 1. Treated students are those with a Moroccan or Turkish migration background who have a high predicted probability of fasting. Exams in 2014–2017 occurred outside Ramadan, while those in 2018–2019 overlapped with Ramadan. The final-exam grade is the standardized average of all central-exam components; the pass rate equals one if a student graduated (including retakes). All specifications include the full set of controls, school–subtrack fixed effects, and exam-year fixed effects, with standard errors clustered at the school–track–subtrack–year level. The horizontal dashed line indicates zero effect

In this section, we present the results for the effect of Ramadan on secondary school outcomes, based on Eq. 1. Figure 3A shows that students with a high predicted probability of fasting experience a significant drop in their central-exam grade in the years when the exams fall during Ramadan. In the three pre-Ramadan-overlap years (2014–2016), the estimated coefficients are small and statistically indistinguishable from zero, with a modest upward tendency that is reversed sharply once Ramadan coincides with the exams. This pattern provides no evidence of differential pre-trends and is consistent with a genuine break in outcomes in 2018–2019. Figure 3B shows a similarly modest and non-significant pattern before 2018, followed by a noticeable decline in the pass rate in the two Ramadan-overlap years.

Table 1 reports the corresponding difference-in-differences estimates from Eq. 2, using the high-probability group as treated. The final-exam grade falls by 5.8% of a standard deviation in years when Ramadan overlaps with the exams (Column 1). The probability of graduating falls by 1.1 percentage points (Column 2), which corresponds to an 8.3% increase in the likelihood of failing to graduate—arguably the more policy-relevant margin, as failing implies repeating a school year or risking dropout. Because graduation depends 50% on the continuous assessment grade (which is not affected by Ramadan), the implied effect on the central-exam component itself would be mechanically larger if graduation were determined solely by the central-exam performance.

**Table 1:** Impact of Ramadan observance on secondary school outcomes

	Final	Pass	Continuous	Improved	Final incl. retakes
	(1)	(2)	(3)	(4)	(5)
Ramadan exam years	−0.058***	−0.011***	0.019	0.007	−0.059***
× Treated (high prob. fasting)	(0.010)	(0.004)	(0.012)	(0.005)	(0.010)
School–subtrack FE	Yes	Yes	Yes	Yes	Yes
Exam year FE	Yes	Yes	Yes	Yes	Yes
All controls	Yes	Yes	Yes	Yes	Yes
Mean outcome before	6.10	86.8%	6.27	26.3%	6.18
Share treated	4.7%	4.7%	4.7%	4.7%	4.7%
Observations	776,219	776,284	776,284	776,284	776,284

Notes: This table reports estimates of β from Eq. 2, comparing students with a high predicted probability of fasting (treated) to students without a Muslim-majority migration background (controls). *Final* is the standardized central-exam grade; *Pass* indicates graduation (including retakes); *Continuous* is the standardized school-based exam grade; *Improved* equals 1 if the student improved their central-exam grade in a retake; *Final incl. retakes* includes retake grades. Differences in observations reflect students who only sat retakes. “Mean outcome before” refers to treated students in pre-overlap years (2014–2017). Controls include student demographics, prior achievement, family composition, migration background, mobility indicators, household income and benefits, and neighbourhood characteristics; Appendix Table B4 shows results when these controls and fixed effects are added stepwise. Standard errors are clustered at the school–track–subtract–year level; robustness to clustering at the school–exam–year level is shown in Appendix Table B5. *p<0.10, **p<0.05, ***p<0.01

Appendix Table B6 shows that the negative Ramadan effects are not an artefact of our preferred ML-based treatment definition. Using either the simpler Moroccan-origin dummy or the continuous fasting-probability measure yields estimates of comparable magnitude and precision, confirming robustness to both binary and continuous alternative classifications of likely fasters. Consistent with this, the event-study plots based on these alternative definitions (Appendix Figs. A10 and A11) display pre-treatment patterns similar to those in Fig. 3, with no evidence of differential trends prior to the Ramadan–exam overlap.

Although pre-trend estimates are small and statistically indistinguishable from zero, visual checks alone provide limited power. We therefore apply the sensitivity framework of Rambachan and Roth (2023), which quantifies how large violations of the parallel-trends assumption—either in levels (magnitude test) or in slopes (smoothness test)—would have to be to overturn our findings. For our main high-compliance specification, Appendix Fig. A14 shows that the estimated Ramadan effects remain negative and economically meaningful under a wide range of plausible pre-trend deviations, and would only disappear under violations substantially larger than those observed in the pre-treatment period. This provides strong reassurance that the main results are not driven by differential pre-trends between treated and control students.

### Robustness and placebos

We first examine whether compositional changes in ability could explain the main results. A concern is that students with a high predicted probability of fasting may differ in academic ability across cohorts, which could generate spurious treatment effects. To assess this, we use the standardized continuous-assessment grade—based on decentralized exams taken over the last 2 to 3 years of secondary school—as a proxy for ability. Appendix Fig. A9 shows no systematic differences in this measure during the Ramadan-overlap years, once we control for standardized end-of-primary-school test scores.^12^ Apart from a small and statistically insignificant deviation in 2014, the pre-treatment years exhibit no signs of differential trends. Column 3 of Table 1 confirms the absence of treatment effects on the continuous-assessment grade, indicating that changes in exam performance are not driven by shifts in student composition. Appendix Table  B7 further confirms the absence of differential compositional changes: a balance test of student, family, and neighbourhood characteristics shows no systematic differences between treated and control students across the pre- and post-overlap periods.

We next examine whether students compensate for lower performance during Ramadan by improving their results on retakes. Column 4 of Table 1 shows only a very small and statistically insignificant increase in the probability that high-probability fasters improve their grade on a retake in the years when Ramadan overlapped with the exams. Consistent with this, the effect on the final grade including retakes (Column 5) is nearly identical to the effect on the initial central-exam grade, indicating that retakes do not offset the negative impact of Ramadan. One possible explanation is that the preparation period for retakes also fell during Ramadan, consistent with Oosterbeek and van der Klaauw (2013), who show that preparing for exams during Ramadan can adversely affect performance.

We further test the robustness of our findings by exploiting within-family comparisons. Using administrative links, we match each exam taker to siblings and cousins who also sat central exams during the 2014–2019 period, which allows us to estimate models with sibling and extended-family fixed effects. These fixed effects absorb all unobserved family characteristics that may jointly affect Ramadan observance and academic performance. Because only a subset of students can be linked to family members in the data, the sample size falls by about 47% when including sibling fixed effects and by about 36% when including extended-family fixed effects.^13^ Running our baseline specification on these restricted samples shows similar effects on exam grades and somewhat larger effects on pass rates, suggesting that any increase in magnitude is attributable to sample selection rather than to the inclusion of fixed effects. Appendix Table B8 reports the sibling and family fixed-effects estimates, which remain negative and significant, confirming that our results are not driven by unobserved family background factors.

As an additional robustness check, we conduct two placebo tests in groups that resemble the treated population socioeconomically but are highly unlikely to observe Ramadan. Using both the within-Turkish and within-Moroccan prediction models, we compute placebo fasting probabilities for students without a migration background from a Muslim-majority country. We then classify non-Muslim students with a high placebo probability as “treated” and re-estimate our specifications. These placebo groups reproduce the socioeconomic characteristics of the true treated group but should not experience any effect of Ramadan. Consistent with this, Appendix Table B9 shows no change in exam performance or pass rates in the Ramadan-overlap years. The absence of any placebo effect strengthens the interpretation that the declines observed among high-probability fasters reflect the impact of Ramadan itself rather than another shock affecting students with a migration background or from disadvantaged environments.

Finally, we assess whether our results depend on the specific threshold used to classify students as having a high probability of fasting. We re-estimate our main specification using a range of alternative cutoffs for the predicted fasting probability. As shown in Appendix Fig. A12, the estimated effects are remarkably stable across thresholds and become larger in absolute value as the threshold increases, consistent with stronger treatment intensity among students most likely to fast. This pattern confirms that our findings are not driven by the arbitrary choice of a particular cutoff.

## Heterogeneity analysis

### Student and school characteristics

**Fig. 4:**
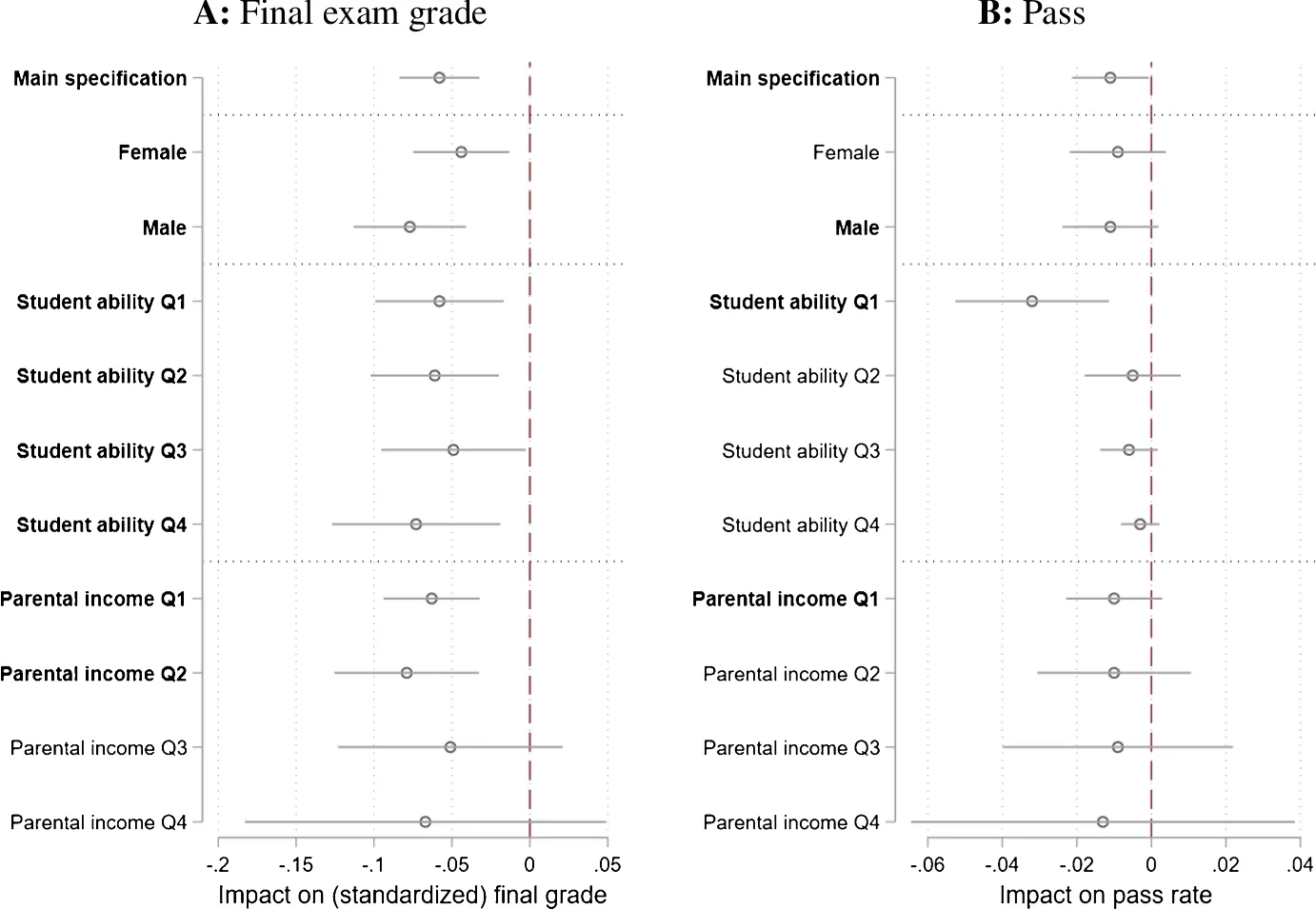
Heterogeneity in the impact of Ramadan on student outcomes, by individual characteristics. **A** Final exam grade. **B** Pass. Notes: Each point shows the estimated coefficient β from Eq. 2, plotted with 99% confidence intervals, for subgroups of students with a high predicted probability of fasting (Moroccan or Turkish background, as classified by the ML model). The first row reproduces the main effect for the full sample. Subsequent rows report estimates separately for female and male students; for quartiles of baseline ability (standardized continuous-assessment grade); and for quartiles of parental income (measured in the year before the exam). Final-exam grade is standardized within track and exam year; the pass indicator equals 1 for graduation (including retakes). Bold labels indicate estimates significant at the 5% level. All specifications include the full set of controls, school–subtrack and exam-year fixed effects, and standard errors clustered at the school–track–subtrack–year level. The horizontal dashed line denotes zero effect

In this section, we examine which students are most affected by Ramadan by exploring heterogeneity along individual characteristics. We first test for gender differences and find broadly similar effects for male and female students, with only slightly larger declines in final-exam grades for males (Fig. 4). We then assess heterogeneity by prior performance, using students’ standardized continuous-assessment grade as our measure of baseline ability. The impact on final-exam grades is remarkably similar across the ability distribution, but effects on pass rates differ sharply: consistent with the structure of the Dutch exam system, the decline in graduation probability is concentrated among students in the lowest ability quartile—those already closest to the pass/fail threshold. For these students, Ramadan-exam overlap increases the probability of failing to graduate by roughly 11%.

**Fig. 5:**
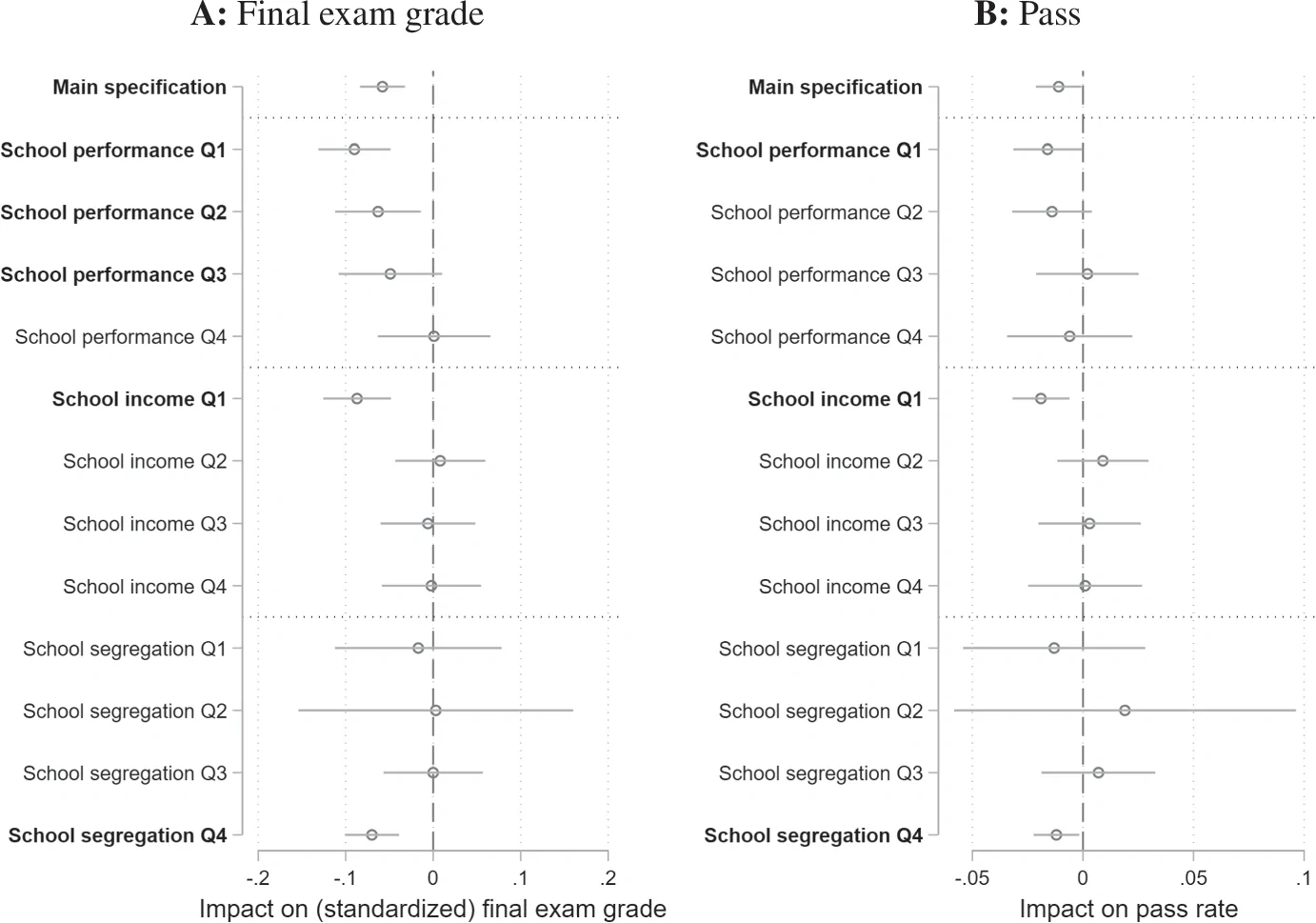
Heterogeneity in the impact of Ramadan on student outcomes, by school characteristics. **A** Final exam grade. **B** Pass. Notes: Each point shows the estimated coefficient β from Eq. 2, plotted with 99% confidence intervals, for subgroups defined by school characteristics. Students are those with a high predicted probability of fasting (Moroccan or Turkish background, ML-based). The first row reproduces the full-sample effect. Subsequent rows show estimates by quartiles of: (i) average exam performance in the school (mean standardized final-exam grade in pre-Ramadan-overlap years); (ii) mean parental income in the school (average income-decile of enrolled students); and (iii) the share of high-probability fasters in the school (our measure of segregation). Final-exam grades and pass indicators are defined as in Fig. 4. Bold labels indicate significance at the 5% level. All specifications include the full set of controls, school–subtrack and exam-year fixed effects, and standard errors clustered at the school–track–subtrack–year level. The dashed line shows zero effect

At the school level, we use the average final-exam grade in pre-Ramadan-overlap years as a proxy for baseline school performance. The pattern mirrors the individual heterogeneity results: the impact of Ramadan is largest in the lowest-performing schools and essentially zero in the top quartile of schools (Fig. 5). For pass rates, the gradient is even clearer—students with a high predicted probability of fasting in the bottom quartile of schools are 11.3% more likely to fail to graduate when exams overlap with Ramadan, whereas no detectable effect appears in the top half of the baseline school performance distribution.

We next examine whether the impact of Ramadan varies by income at either the student or school level. Individual family income does not meaningfully moderate the effect (Fig. 4); students from higher- and lower-income households experience similar declines when exams overlap with Ramadan. In contrast, school-level income shows substantial heterogeneity. The entire effect on both final grades and pass rates is concentrated in schools in the lowest income quartile, while no significant impact is detected in schools with above-median income levels (Fig. 5). This divergence suggests that school-level resources or support environments, rather than individual family income, shape the extent to which fasting students can absorb the challenges posed by Ramadan during exam periods. We note, however, that baseline school performance and school-level income are strongly correlated in our data, making it difficult to disentangle their separate contributions.^14^

We also investigate whether the effect of Ramadan varies with the degree of school segregation, measured as the share of students with a high predicted probability of fasting in pre-Ramadan-overlap years. The negative impact on both final grades and pass rates is concentrated entirely in the 25% most segregated schools (Fig. 5); in the remaining schools, we detect no significant effect. This pattern may reflect self-selection—students who are most likely to fast tend to cluster in the same schools—but it may also indicate peer reinforcement of fasting behaviour, whereby students adhere more strictly to Ramadan practices when surrounded by more observant peers.

To distinguish these channels, we exploit variation at the cohort level within schools. A cohort is defined as students in the same subtrack, track, school, and exam year. Appendix Fig. A13 shows substantial within-school, across-cohort variation in the share of high-probability fasters, even after accounting for the more deterministic selection at the school level. Cohort composition is therefore less subject to sorting and provides a cleaner source of variation for testing peer effects. When we interact our treatment indicator with the cohort-level share of likely fasters, point estimates suggest that the negative impact of Ramadan is larger in cohorts with more observant peers (Appendix Fig. A15). This pattern is consistent with peer reinforcement effects and suggests that school-level heterogeneity is not solely driven by selection into schools. We further note that segregation is itself strongly correlated with both income and baseline performance in our data, making it difficult to separately identify its role from broader school disadvantage.^15^ Appendix Fig. A15 exploits within-school cohort variation to provide a partial test, though we acknowledge that the coefficients of interest in this specification do not reach conventional significance levels, and the evidence should therefore be interpreted cautiously.

### Effect by type of exam taken

To assess whether Ramadan affects some exams more than others, we re-estimate our main specification using subject-level central-exam grades (e.g. mathematics, English, biology) rather than the overall final exam grade. The pattern of results closely mirrors the baseline: students with a high predicted probability of fasting perform systematically worse when exams fall during Ramadan, although the magnitudes are slightly smaller in the subject-level specification (Appendix Table B10). This attenuation is expected given (i) the greater weight placed on pre-university students—who take more exams and are less affected—and (ii) the richer set of fixed effects needed to account for the exact timing of each subject exam.^16^

The subject-level data also allow us to group exams according to their cognitive demands. Appendix Table B11 shows that memory-based subjects (history, geography, biology) exhibit a decline of about 4.2% of a standard deviation, whereas language exams (Dutch, English, German, French reading comprehension) show a smaller effect of around 2.4% of a standard deviation. The largest impact appears for mathematics, where grades fall by 8.9% of a standard deviation, more than twice the size of the effect for memory-based subjects.

These patterns shed light on potential mechanisms. The larger deterioration in memory-heavy subjects is consistent with experimental evidence that sleep deprivation disproportionately impairs memory consolidation and attentional processes relative to reasoning and crystallized intelligence (Killgore 2010; Lim and Dinges 2010; Van Dongen et al. 2003). Ramadan observance is well-documented to disrupt sleep through shifts in eating schedules around pre-dawn and post-sunset meals, which would be consistent with this pattern. However, we cannot rule out other biological channels. Fasting-induced hypoglycaemia, for instance, could also differentially affect memory-dependent tasks, as glucose availability is known to support hippocampal function and memory encoding. The substantial decline in mathematics performance—a subject requiring sustained reasoning rather than recall—further suggests that fasting-related fatigue or reduced concentration plays an additional role beyond sleep disruption alone. In short, the subject heterogeneity is consistent with a mix of sleep and fasting channels, but we are cautious about attributing it to any single physiological pathway. The next subsection examines the timing of exams within days and weeks to further distinguish between these mechanisms.

## Mechanisms and policy implications

### When do Ramadan-related performance losses emerge?

Ramadan observance may affect exam performance through multiple channels. Beyond fasting from sunrise to sunset, sleep schedules often shift around pre-dawn and post-sunset meals, and time spent on family or religious activities may increase. While our empirical setting does not allow us to cleanly disentangle these mechanisms, we can examine when within the exam period performance decreases are most pronounced.

We begin by testing whether effects accumulate as Ramadan progresses. If cumulative sleep loss were the primary channel, exam performance should worsen toward the end of the month (Van Dongen et al. 2003). Conversely, if the adjustment to fasting is initially most challenging, declines might be greatest during the first days. Appendix Fig. A17 shows estimated effects by week of Ramadan. Effects are negative throughout the period when exams overlap with Ramadan, but we find no systematic deterioration over successive weeks, nor a spike in the initial days.^17^ Given the relatively short 2-week overlap in our setting and limited precision at this level of disaggregation, we interpret these patterns cautiously. Nevertheless, they indicate that the decline in performance does not meaningfully accumulate across the fasting period.

Appendix Fig. A16 shows the number of exam takers and exams by timing and migration background across all years in our sample. The distribution of morning and afternoon exams is broadly similar for high-compliance students and other students and stable across years, confirming that treated and control students face comparable exam schedules with no differential sorting into exam timing around the Ramadan-overlap years.

We next examine variation within the school day. On some days, students sit both a morning and an afternoon exam, creating differential exposure to prolonged fasting and cognitive effort. Appendix Table B12 first reports a simpler specification interacting the DiD estimate with an afternoon dummy, irrespective of whether one or two exams occur on the same day. The overall Ramadan effect is −0.040 standard deviations, and the interaction with the afternoon dummy is small and statistically indistinguishable from zero (+0.010), indicating that morning and afternoon exams are similarly affected on average when considered in isolation. This aggregate comparison, however, masks important heterogeneity that only emerges when accounting for the number of exams on the same day.

**Fig. 6:**
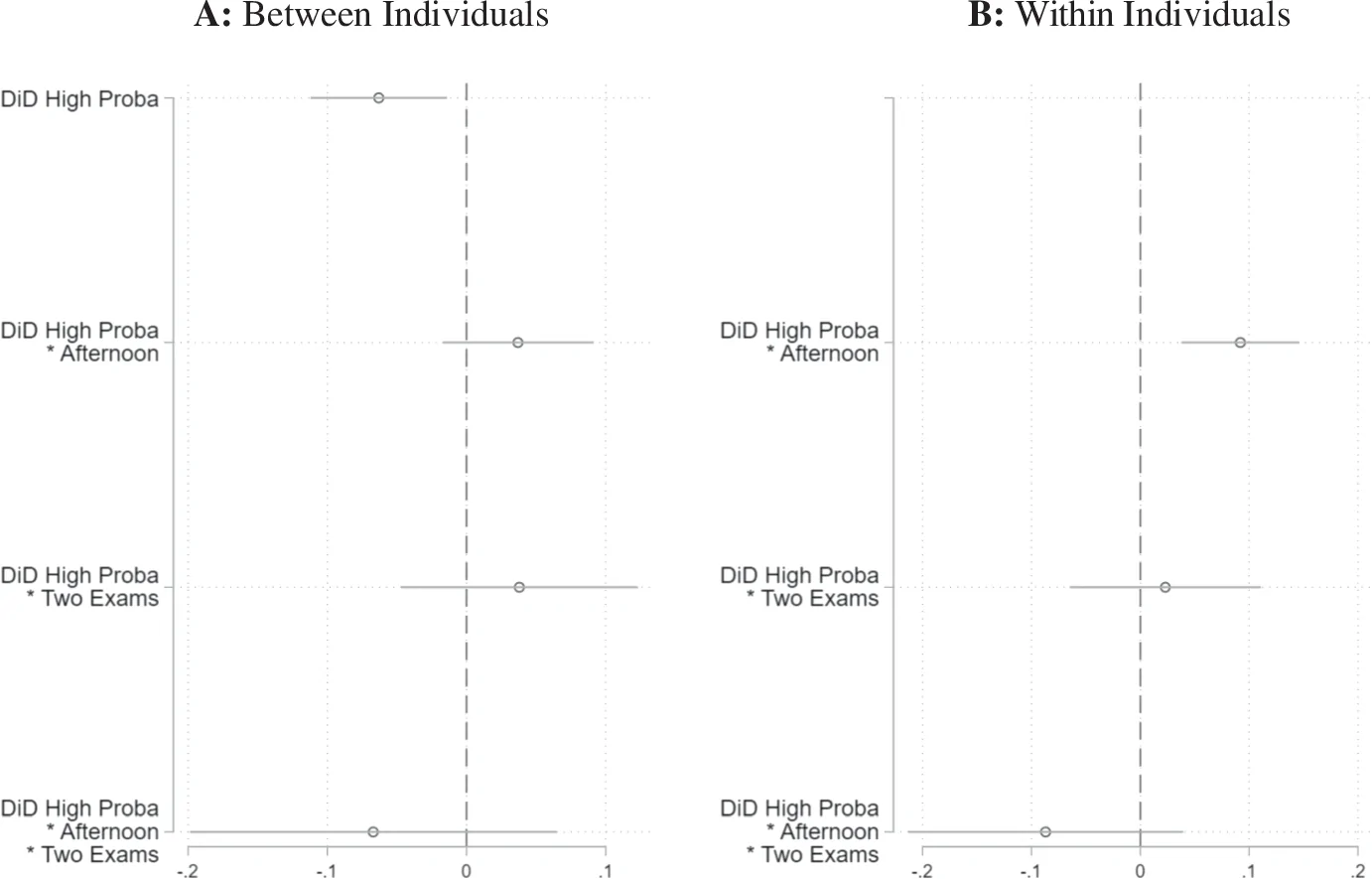
Ramadan effects on exam performance by number of exams taken on the same day. **A** Between individuals. **B** Within individuals. Notes: This figure plots point estimates and 99% CIs from Eq. (C2) in Appendix C.5, estimated with (right) and without (left) individual fixed effects using subject-level data. The dependent variable is the standardized exam grade. $DiD \ High \ Proba$ equals 1 for exams taken during Ramadan by students with a high predicted probability of fasting; *Afternoon* equals 1 for exams held in the afternoon; and TwoExams equals 1 on days when a student sits both a morning and an afternoon exam. All specifications include the controls from Table 1 and school–track–subject and exam-timing fixed effects. Standard errors clustered at the school–track–subject–year level

Figure 6 provides a more refined picture. About 18% of exams in our sample fall on two-exam days, split roughly equally between morning and afternoon sittings.^18^ The results suggest that afternoon exams are less affected by Ramadan than morning exams when taken in isolation, but that this advantage is largely undone when a morning exam has already been sat on the same day. It is therefore specifically the combination of a morning exam followed by an afternoon exam on the same fasting day that creates the most demanding conditions—not afternoon exams per se. We treat these timing results as exploratory given power limitations at this level of disaggregation, but they point to a concrete and targeted policy implication discussed below.

We also assess broader time-pressure mechanisms. If family and social obligations increase during Ramadan, thereby reducing available preparation time, then performance losses should be larger for students with more extensive family networks nearby. Appendix Table B13 exploits administrative links to relatives and shows only marginal differences with no systematic pattern, suggesting that reduced preparation time is, at best, a secondary channel.

Taken together, the evidence points to a mechanism operating primarily during the fasting school day. Afternoon exams appear less affected by Ramadan than morning exams when taken in isolation, but this advantage is eliminated when students also sit a morning exam on the same day, suggesting that it is sustained cognitive effort under fasting conditions that drives the largest losses rather than the time of day per se. We emphasize that these timing results are informative but exploratory due to power limitations at the subject level, and we remain cautious about attributing them to a specific physiological pathway.

### Policy implications

Adverse exam-day conditions can have lasting consequences. Students who miss the pass threshold must repeat the year or move into adult education. Appendix Table B14 shows that when Ramadan overlaps with exams, high-probability fasters are 12.4% more likely to repeat the final year. For those aged 18 or older—who can legally exit education entirely—Table 2 shows a 22.5% increase in dropout. These are meaningful losses in human capital: an additional year of schooling delays graduation, while dropping out can permanently limit labour-market opportunities.

**Table 2:** Impact of Ramadan on longer-term outcomes, by age at exam

	Long-term outcomes					
	Younger than 18			18 or older		
	Grade	Adult	Drop	Grade	Adult	Drop
	retention	education	out	retention	education	out
	(1)	(2)	(3)	(4)	(5)	(6)
Ramadan exam years	0.006**	0.004*	0.001	0.008	0.000	0.017**
× Treated (high prob. fasting)	(0.003)	(0.002)	(0.004)	(0.005)	(0.008)	(0.008)
School–subtrack FE	Yes	Yes	Yes	Yes	Yes	Yes
Exam-year FE	Yes	Yes	Yes	Yes	Yes	Yes
All controls	Yes	Yes	Yes	Yes	Yes	Yes
Share treated	5.32%	5.32%	5.32%	3.34%	3.34%	3.34%
Mean outcome	5.86%	2.18%	4.26%	4.68%	14.5%	7.59%
Observations	536,927	536,927	536,927	238,111	238,111	238,111

Notes: This table reports estimates of β from Eq. 2, using long-term education outcomes 1 year after the exam. Treated students are those with a high predicted probability of fasting (Moroccan or Turkish background) based on our machine-learning model. The sample is split by whether the student is younger or older than 18 on September 1 following the exam. *Grade retention* equals 1 if the student repeats the final year; *Adult education* equals 1 if the student enrolls in adult education; and *Drop out* equals 1 if the student does not enroll in any education the following year. All regressions include the full control set from Appendix Table B6. Standard errors, in parentheses, are clustered at the school–track–subtrack–year level. *p<0.10, **p<0.05, ***p<0.01

The timing evidence should be interpreted cautiously and does not rule out broader behavioural channels—such as reduced learning time during Ramadan (Dasgupta and Datta 2024). That said, it points to two concrete and targeted scheduling adjustments that could meaningfully reduce the disadvantage faced by observant students when overlap is unavoidable: preferring afternoon slots for individual exams during Ramadan, and avoiding the scheduling of a morning exam on the same day as an afternoon exam. The first reduces exposure to the most cognitively demanding part of the fasting day; the second ensures the afternoon advantage is not undone by prior cognitive effort. Neither adjustment requires religious exemptions or changes to students’ devotional practice.

This small scheduling consideration would help ensure that high-stakes exams continue to evaluate what students know, rather than how well they can endure fasting during a long school day.

## Conclusion

This paper provides the first causal evidence on how the overlap between Ramadan observance and high-stakes national exams affects the educational outcomes of Muslim-origin students in a Western European context. Exploiting year-to-year variation in the timing of Ramadan relative to nationwide secondary-school exams in the Netherlands, and combining detailed administrative microdata with survey-based measures of religious practice, we estimate the impact of fasting during exam days on performance and graduation outcomes.

We document sizable negative effects for students with a high predicted probability of observing the fast. Their average standardized central-exam grades fall by roughly 0.06 standard deviations when Ramadan overlaps with the exam period, and their probability of obtaining a diploma declines accordingly. These magnitudes are comparable to the impacts of environmental shocks such as heat waves (1 SD increase in temperature, Park, 2022) or severe air pollution (1.5 SD increase, Ebenstein et al., 2016), highlighting that the timing of religious rituals can meaningfully shape academic performance under modern institutional arrangements.

The consequences are particularly pronounced for students already at the lower end of the achievement distribution. Because many Muslim-origin students in the Netherlands—particularly those with a Moroccan or Turkish migration background—enter secondary school with pre-existing socioeconomic disadvantages, the overlap between Ramadan and national exams risks widening educational inequalities for these groups. We estimate that the achievement gap relative to students without a Muslim-majority migration background rises by roughly 20%,^19^ with even larger effects in more religiously segregated school environments. Among those aged 18 or older, who may legally leave school, dropout rates increase substantially when Ramadan falls during exams, suggesting that short-run performance losses may carry long-run consequences.

Analysis of potential mechanisms indicates that timing within the fasting day matters: afternoon exams are less affected by Ramadan than morning exams when taken in isolation, but this advantage is eliminated on days when students also sit a morning exam. This pattern is consistent with accumulated fatigue and limited opportunities to rest during fasting days. While this evidence is exploratory, it suggests that avoiding two-exam days when Ramadan overlaps with exams could be a practical and low-cost way to mitigate harm.

More broadly, our findings underscore the importance of information and awareness. Recent experimental evidence shows that when individuals learn that fasting can impair performance, many adjust their behaviour accordingly (Wang et al., 2023). By quantifying the consequences of fasting during decisive educational transitions, our study can help students, families, and educators better weigh religious obligations against high-stakes academic demands. When formal accommodations are politically or logistically infeasible, transparent communication of these trade-offs may itself reduce harm—while respecting the autonomy and diversity of religious practice.^20^

Taken together, the evidence demonstrates that the collision of a major religious practice with a rigid secular institution can unintentionally deepen educational disadvantage. Thoughtful scheduling adjustments—and improved awareness of existing religious allowances—can help reduce these inequalities, supporting the academic success of affected students without diminishing the significance of their religious commitments.^21^

## Supplementary Information

Below is the link to the electronic supplementary material.

Supplementary file 1 (pdf 5750 KB)

## Data Availability

The analysis uses non-public administrative microdata from Statistics Netherlands (CBS). These data cannot be shared by the authors: access is granted by CBS to accredited institutions under contract and after signing a confidentiality agreement, and all output is subject to CBS disclosure control. Details of the registers used are given in Appendix C. Information on obtaining access is available at CBS Microdata Services. The SIM (*Survey Integratie Minderheden*) waves are distributed by DANS and are similarly available to accredited researchers. Mosque location data were collected from moskeewijzer.nl. All Stata and R code required to reproduce the results from the source data is available from the corresponding author on request. The empirical analysis was conducted in the CBS microdata environment using Stata (MP) and R 4.4.3; the HonestDiD sensitivity analysis uses the honestdid package.

## Footnotes

1 There has been attention on this topic in several countries, such as the UK (“Popular exams in UK to be rescheduled to avoid Ramadan”, *The Guardian*), the USA (“Testing and Fasting”, *Inside Higher Ed*), France (“*Jeûner pendant le bac: le défi des lycéens qui font le Ramadan*”, *France 24*), Germany (“Hungry Students? Postponed Exams? Ramadan In German Schools”, *Worldcrunch*), Italy (“*La Maturità incrocia il Ramadan*”, *Corriere della Sera*), and the Netherlands (“*Vasten en eindexamens, gaat dat wel samen?*”, *NOS*).

2 See Adolphus et al. (2016) and Wittbrodt and Millard-Stafford (2018) on cognitive effects of fasting; Bahammam (2003) and Bahammam et al. (2013) on sleep disruption; and Tian et al. (2011), Ghayour Najafabadi et al. (2015), and Yasin et al. (2013) for contrasting laboratory findings.

3 Among these students, 95.1% (Moroccan) and 92.7% (Turkish) were second-generation migrants (born in the Netherlands with at least one parent born in the origin country); 3.2% (3.6%) were first generation; and 1.7% (3.7%) were third generation (born in the Netherlands to Dutch-born parents but with at least one grandparent born in Morocco or Turkey). Most parents in these communities arrived during the early-1960s to late-1980s labour-migration wave and subsequent family-reunification period.

4 We verified that including students from all Muslim-majority origins and classifying them as treated based solely on country of origin produces estimates nearly identical to our main results. Given the very small size of these groups and substantial heterogeneity in religiosity (including sizeable non-Muslim minorities), using country of origin alone would add noise without additional identifying variation. We therefore focus on Moroccan- and Turkish-origin students for credible treatment assignment.

5 The standardized end-of-primary-school assessment was not mandatory during our cohorts, and a small share of pupils took an alternative test. For all cohorts in our dataset (who took the CITO between 2008 and 2015), the CITO test never overlapped with Ramadan: it was held in February (2008–2012) or April–May (2013–2015), whereas Ramadan occurred between June and September. We therefore use the CITO score when available and show that excluding this control leaves all main results unchanged.

6 Two clarifications regarding timing relative to Ramadan in other years: (i) June retakes overlapped with Ramadan in 2015 (for 1 day), 2016, and 2017, but because students may resit at most one exam, this had negligible implications for final grades or graduation rates; and (ii) continuous school assessments, scheduled by schools throughout the year, occasionally overlapped with Ramadan in pre-2019 years, but these contribute only a very small fraction to the multi-year continuous-assessment grade used in our analysis.

7 Original Dutch text available at the website of the Dutch Parliament.

8 Appendix C provides the precise CBS classification rules.

9 This level best captures correlated shocks among students sharing teachers, peers, and school-track contexts. Results are robust to clustering at broader levels such as the school.

10 We assume that individuals most likely to fast under normal circumstances are also the most likely to do so during high-stakes exams. If anything, religious observance may be stronger during Ramadan exams, making our treatment definition conservative.

11 With around 2000 observations per group, methods that rely on large-sample feature extraction (e.g. deep learning) risk severe overfitting.

12 Using the standardized end-of-primary-school test score itself as the outcome reveals a small difference in 2014, which underscores the importance of controlling for prior achievement. Conditional on this measure, however, we find no evidence of differential selection into exam-taking across cohorts.

13 Family links are constructed by matching students to siblings and cousins observed in BRP and exam registers. Link rates are lower for students whose parents or grandparents do not appear in the population registers, especially among first-generation migrants.

14 Among students in the bottom quartile of baseline school performance, 41% are also in the lowest income quartile, and 64% are in the bottom half of the income distribution. Conversely, among students in the lowest income quartile of schools, 41% are also in the bottom quartile of baseline school performance, and 70% are in the bottom half. The effects attributed to either dimension should therefore be interpreted as reflecting a broader school disadvantage environment rather than distinct mechanisms.

15 Among students in the most segregated schools (top quartile of the fasting-probability distribution), 50% are in the lowest income quartile, and 40% are in the lowest baseline performance quartile. Among students in the lowest income quartile of schools, 49% are in the most segregated quartile; among students in the bottom quartile of baseline school performance, 39% are in the most segregated quartile.

16 We standardize subject outcomes at the subject–track–year level, rather than at the track–year level as in the baseline. A small number of subjects are taken only by specific tracks, generating compositional differences in the subject-level design. The corresponding regression specification is provided in Eq. (C1) in Appendix C.5

17 A small number of exams in 2018 occurred in the week just before Ramadan; coefficients for this pre-overlap window are close to zero, suggesting minimal anticipatory changes in sleep or eating routines.

18 The coefficients in the figure are cumulative and must be read additively relative to the baseline of a morning exam on a single-exam day. Afternoon exams on single-exam days are somewhat less affected than morning single-exam days (DiD × Afternoon is positive); morning exams on two-exam days show a similar effect to the baseline (DiD × Two Exams ≈ 0); but the triple interaction (DiD × Afternoon × Two Exams) is negative, meaning that afternoon exams taken after a morning exam on the same day are associated with substantially larger losses—partially offsetting the otherwise smaller afternoon effect. While imprecisely estimated, this pattern is consistent across both specifications.

19 In non-overlap years (2014–2017), the gap in pass rates between students without a Muslim-majority migration background and high-compliance students was approximately 5.8 percentage points (92.7% vs. 86.9%). During Ramadan-overlap years (2018–2019), this gap widened to approximately 7.0 percentage points (92.6% vs. 85.6%), while the pass rate for students without a Muslim-majority migration background remained virtually unchanged (92.7% vs. 92.6%). This raw comparison yields a somewhat larger estimate than our regression-based figure, as the latter controls for student and school characteristics.

20 Some Islamic authorities, including in the UK and Egypt, have already issued rulings permitting exemptions for students whose academic performance would be significantly affected by fasting. See Leeds Grand Mosque (fasting-related-fatwas) and Dar al-Ifta al-Missiyyah (ruling 1878).

21 The contrast with the parliamentary exchange quoted at the opening of this paper is instructive: in 2023, a parliamentary majority successfully pressured the Ministry of Education to extend the primary-school standardized assessment window by 1 week to accommodate carnival celebrations in the south of the Netherlands (*NOS*, 11 September 2023). Scheduling flexibility, it appears, is feasible when sufficient political will exists.

## Declarations

### Use of generative AI

The authors used Claude (Anthropic) for language editing and proofreading of the manuscript. The authors reviewed and edited all content and take full responsibility for the publication.
