# Supplementary material for: The fast and the studious? Ramadan observance and student performance
Source: J Popul Econ. 2026 Oct 6;39(4):70. doi: 10.1007/s00148-026-01204-9 (PMC13642512; doi:10.1007/s00148-026-01204-9)
Supplement: Supplementary file 1 [file 148_2026_1204_MOESM1_ESM.pdf]

# Online Appendix

## The fast and the studious?
## Ramadan observance and student performance

Kyra Hanemaaijer Olivier Marie Marco Musumeci

Kyra Hanemaaijer, University of Gothenburg

Olivier Marie, Erasmus University Rotterdam, TI, CEPR, IZA, and CESifo

Marco Musumeci, University of Padova

Corresponding author: Olivier Marie, marie@ese.eur.nl

*Journal of Population Economics*

This online appendix supplements the article "The fast and the studious? Ramadan observance and student performance." It contains additional figures (Appendix A), additional tables (Appendix B), and details on data construction, the SIM survey, and supplementary regression specifications (Appendix C). It is not intended to be read independently of the main article, to which all references in the text refer.

# A Additional figures

**Figure A1:** Municipal distribution of Moroccan- and Turkish-origin residents and mosque locations

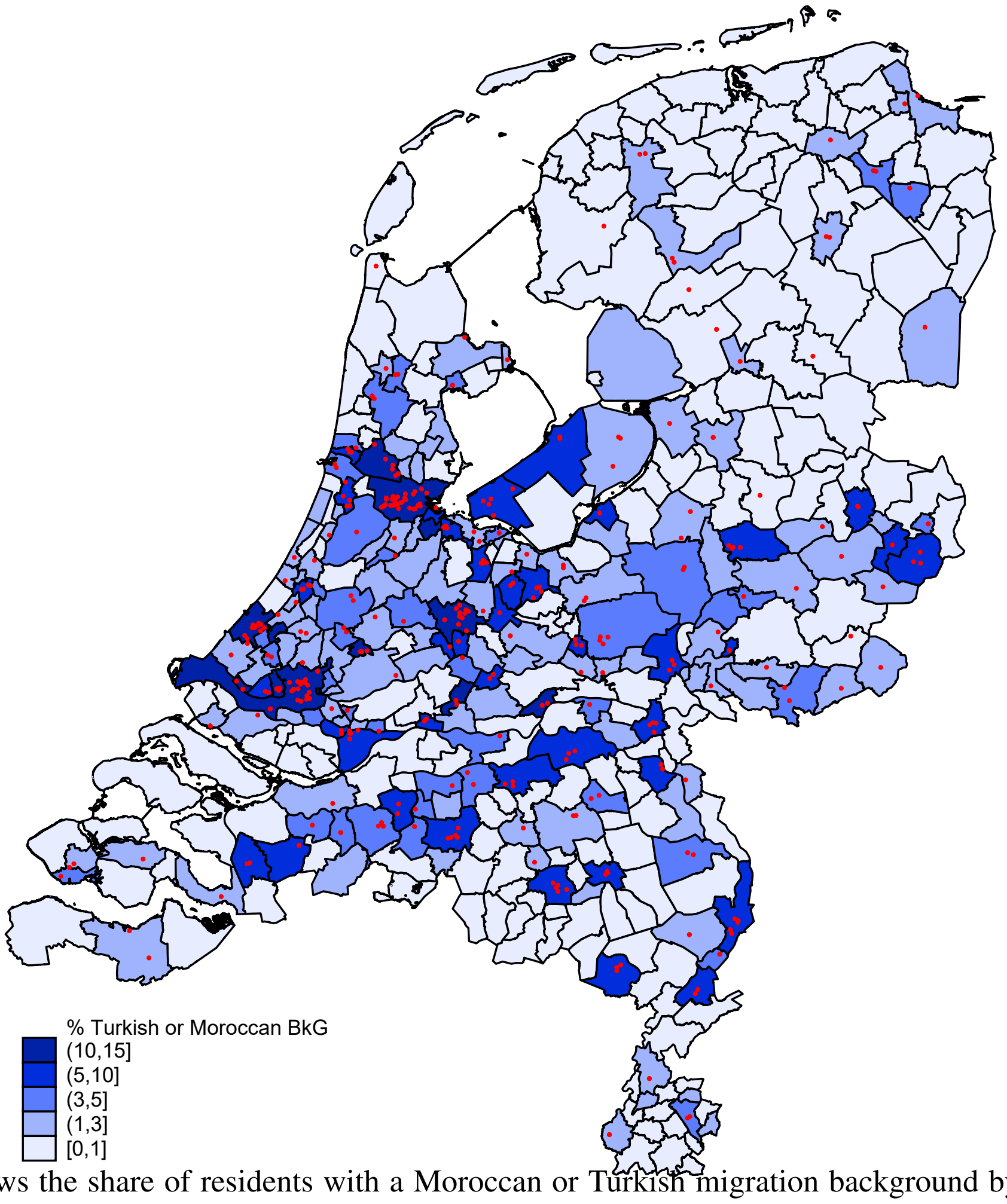


Notes: Map shows the share of residents with a Moroccan or Turkish migration background by municipality in 2017. Red dots indicate locations of mosques where the sermon language is Turkish or Moroccan Arabic. Source: CBS Statline; Moskeewijzer.

**Figure A2:** Dutch education system

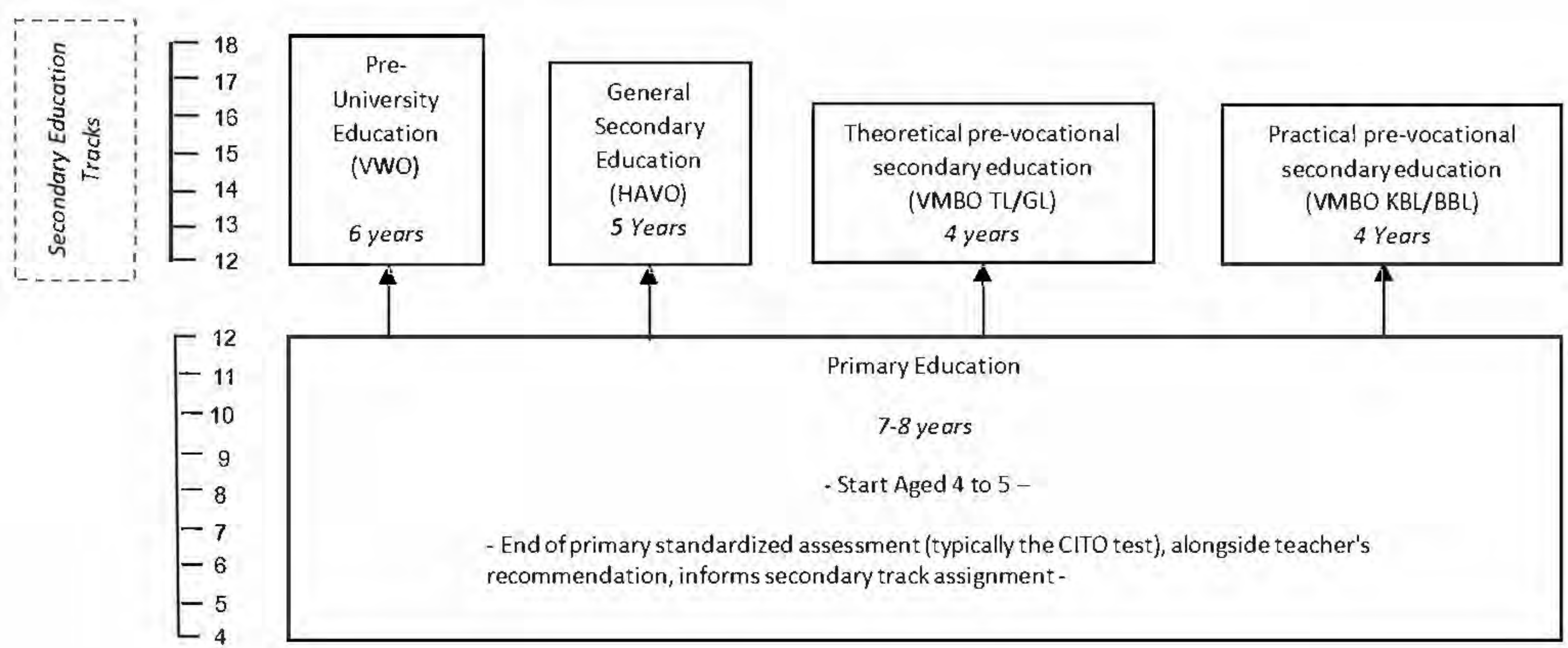


Notes: Detailed graphical overview of the education system from primary education to the end of secondary education in the Netherlands.

**Figure A3:** Graphical representation of overlap between final exams in secondary school and Ramadan.

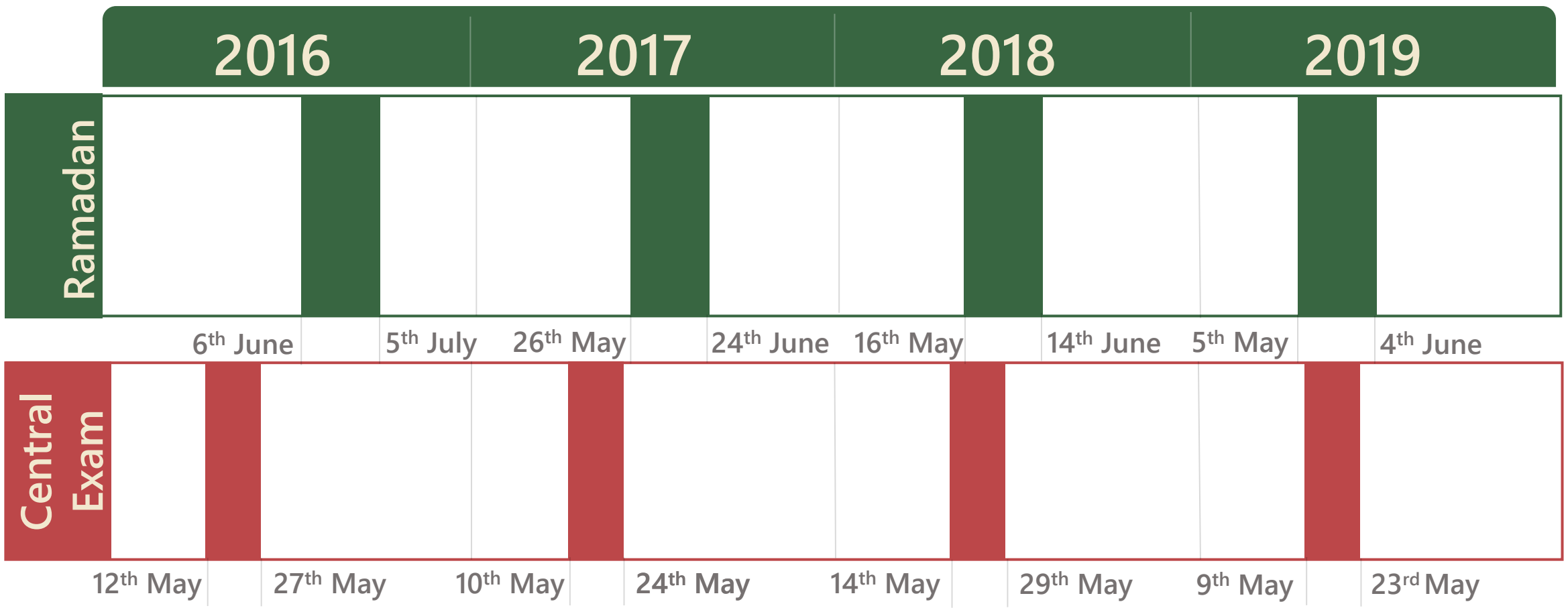


Notes: Graphical representation of the overlap between the central exams and Ramadan. In the years before and including 2017, there is no overlap between the two, even if in 2017 they are very close. From 2018, the central exam and Ramadan occur in the same period (only partially in 2018). Source: Examenblad.nl, https://www.examenblad.nl/.

**Figure A4:** Distribution of characteristics of students by migration background

**A:** Standardized final exam grade

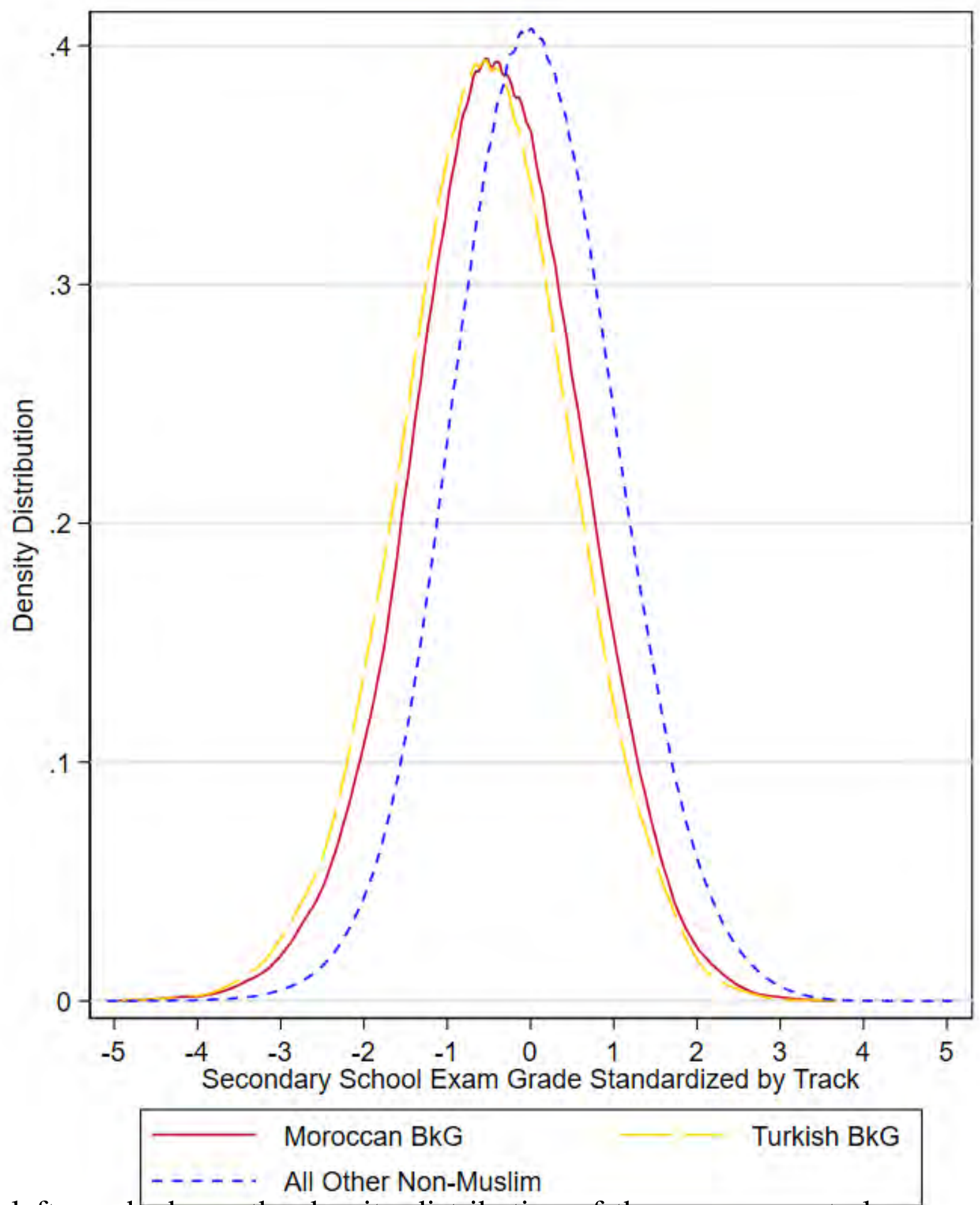


**B:** Other characteristics

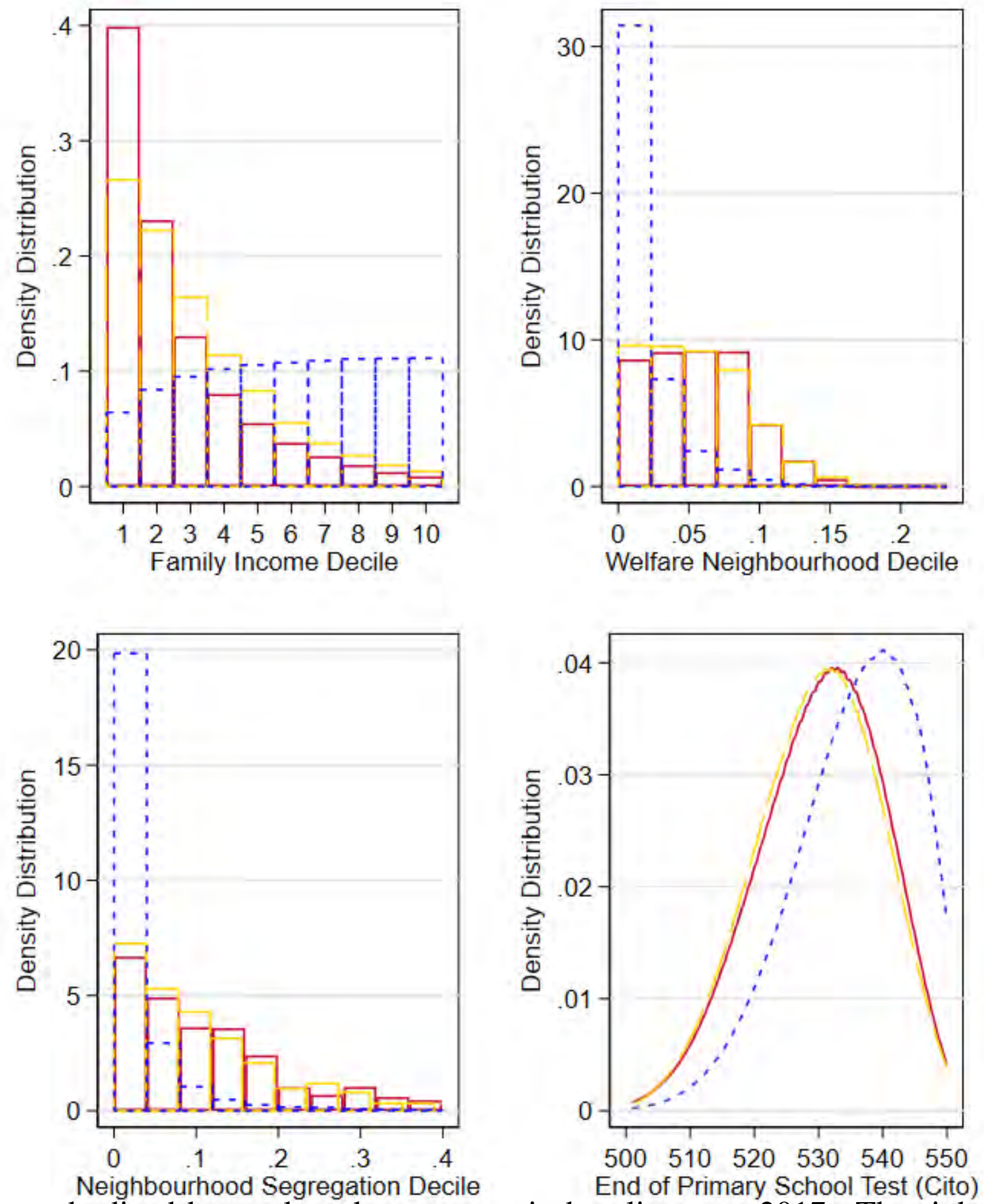


Notes: The left graph shows the density distribution of the average central exam grade standardized by track and exam year in baseline year 2017. The right graph plots the density distribution of family income divided in deciles, the share of a neighbourhood using welfare (*bijstand*), the share of a neighbourhood with a Moroccan migration background and the grade of the end of primary school test (standardized by year). The distributions are given for students with a Moroccan migration background (red solid line), Turkish migration background (yellow long-dashed line) and a non-Muslim migration background or no migration background (blue short-dashed line).

**Figure A5:** Fasting behavior of SIM respondents with a Moroccan or Turkish BkG by age (SIM survey 2006-2020)

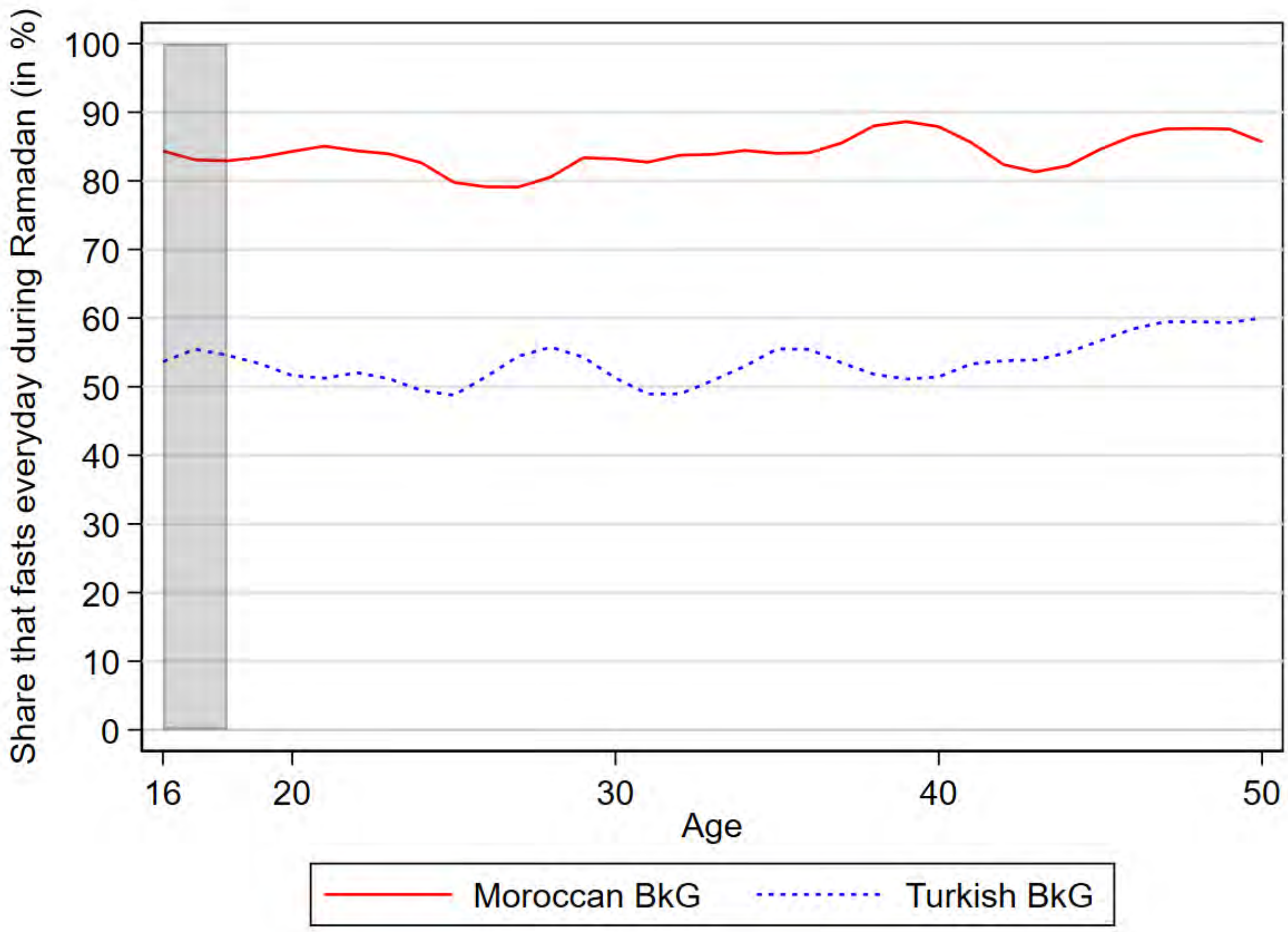


Notes: Graphical representation of the average share of respondents with a Moroccan migration background (red solid line) or Turkish migration background (blue dashed line) that report to fast everyday during Ramadan by age at the time of the survey. The grey area indicates the age of exam takers. Source: SIM (*Survey Integratie Minderheden*) data on 2006, 2011, 2015 and 2020.

**Figure A6:** Event–study estimates for student characteristics (treated: high predicted Ramadan fasters)

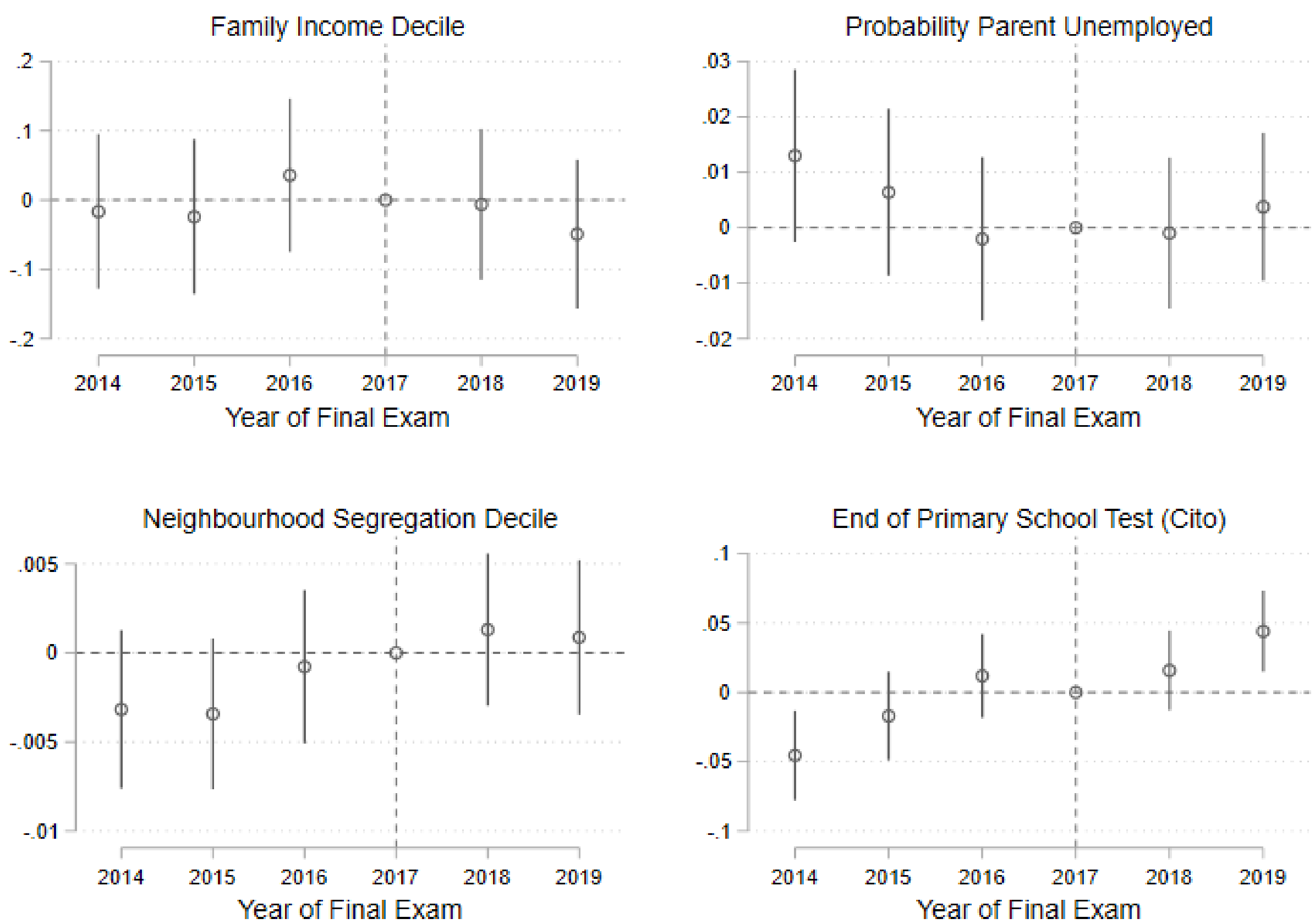


Notes: Each panel plots event–study coefficients and 99% confidence intervals from an extension of equation (1), where the dependent variables are student and neighbourhood characteristics rather than exam outcomes. Treated students are those with a Moroccan or Turkish migration background who have a high predicted probability of fasting. Characteristics include: (i) parental income decile, (ii) indicator for parental unemployment, (iii) neighbourhood concentration of Moroccan- or Turkish-origin residents (deciles), and (iv) standardized primary-school test score (CITO). All specifications include the full control set from Table B6, except when a control overlaps with the dependent variable. School–subtrack and exam-year fixed effects are included, with standard errors clustered at the school–track–subtrack–year level. Flat pre-trends and small, statistically insignificant coefficients in the Ramadan-overlap years indicate no differential selection or composition shifts by treatment status over time. The horizontal dashed line marks zero effect.

**Figure A7:** Penalized logit model coefficients: Moroccan BkG

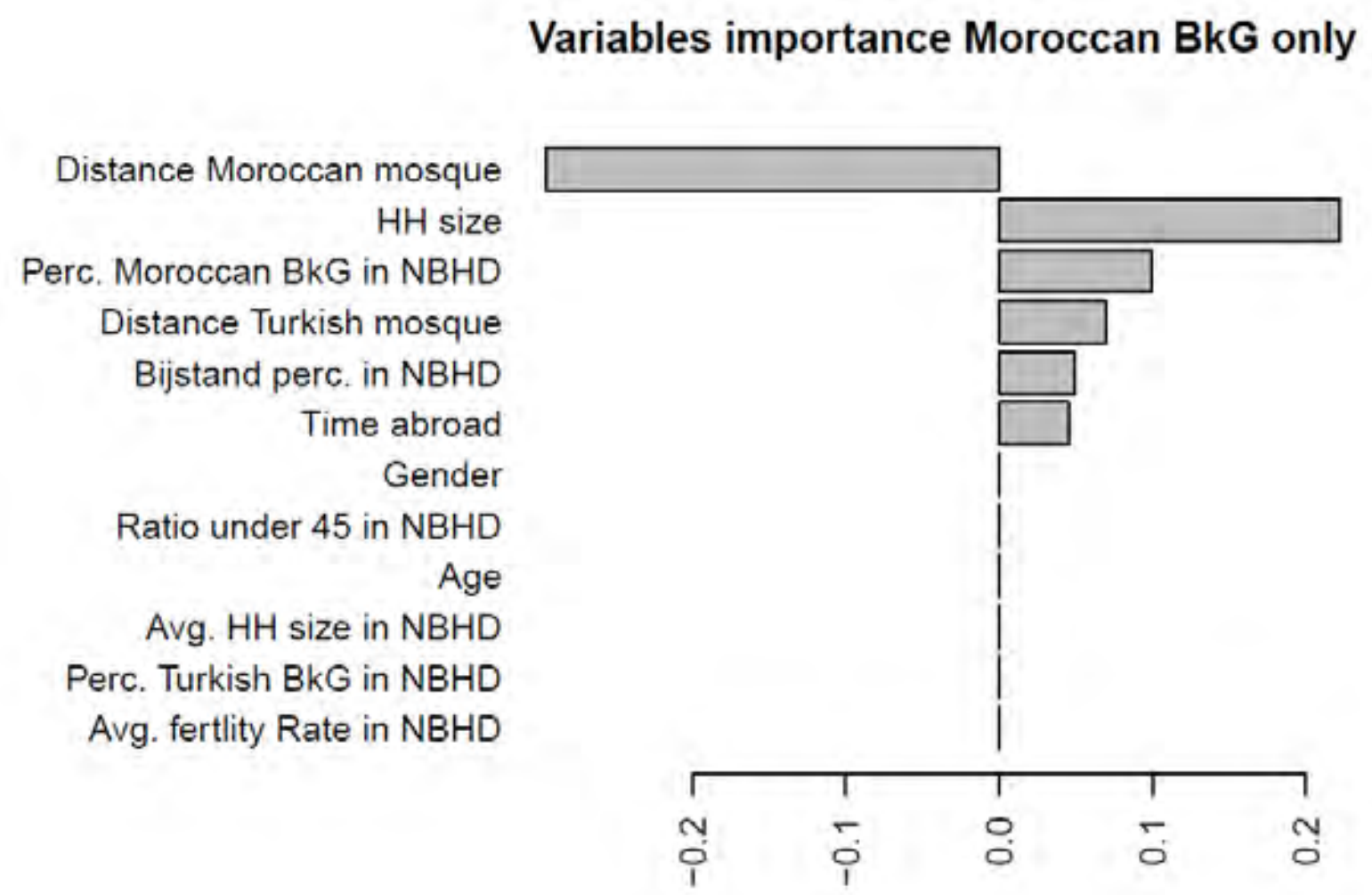


Notes: Importance of variables for prediction of Ramadan compliance probability as predicted with a penalized logit ML model including only SIM respondents with a Moroccan migration background. Variables used in the model are the share of the respondent's neighbourhood having a Moroccan neighbourhood or Turkish neighbourhood, age of the respondent, time spent abroad, gender of the respondent, average household size in the respondent's neighbourhood, distance to Moroccan or Turkish mosque, share of the neighbourhood using welfare (*bijstand*), fertility in the neighbourhood and the share of the respondent's neighbourhood below 45 years old.

**Figure A8:** Penalized logit model coefficients: Turkish BkG

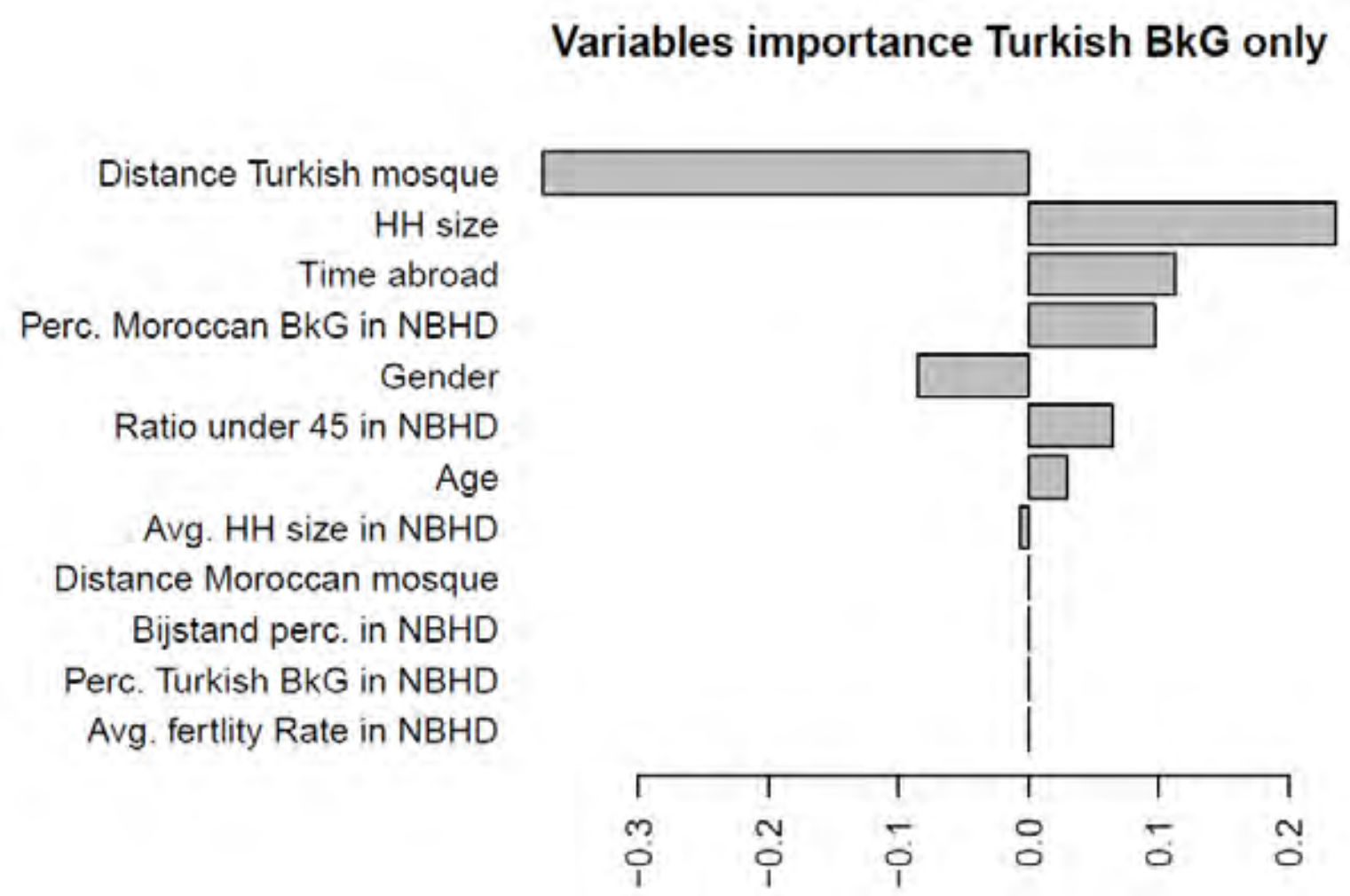


Notes: Importance of variables for prediction of Ramadan compliance probability as predicted with a penalized logit ML model including only SIM respondents with a Turkish migration background. Variables used in the model are the share of the respondent's neighbourhood having a Moroccan neighbourhood or Turkish neighbourhood, age of the respondent, time spent abroad, gender of the respondent, average household size in the respondent's neighbourhood, distance to Moroccan or Turkish mosque, share of the neighbourhood using welfare (*bijstand*), fertility in the neighbourhood and the share of the respondent's neighbourhood below 45 years old.

**Figure A9:** Impact of Ramadan on *continuous exam grades* of students with a high fasting probability

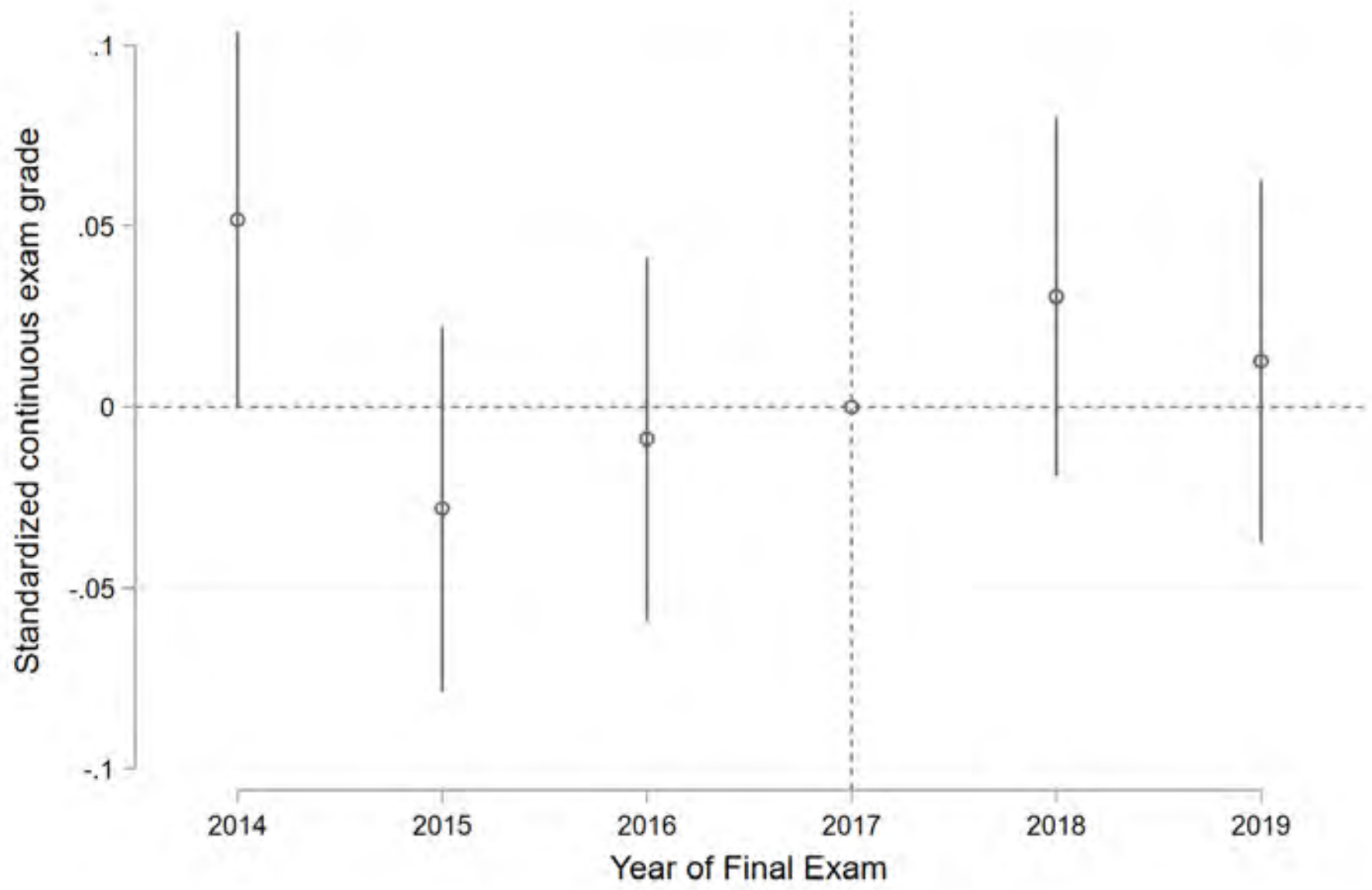


Notes: The figure plots event-study coefficients ($\beta_t$) and 99% confidence intervals from equation (1) in the main text, with the standardized continuous (school) exam grade as the dependent variable, controlling for the standardized end-of-primary-school test score. Treated students are those with a Moroccan or Turkish migration background and a high predicted probability of fasting. 2017 is the reference year. All other controls, fixed effects and clustering (school–track–subtrack–year) are as in Figure 3 in the main text. The horizontal dashed line marks zero effect.

**Figure A10:** Ramadan effects for students with a Moroccan migration background

**A:** Standardized final-exam grade

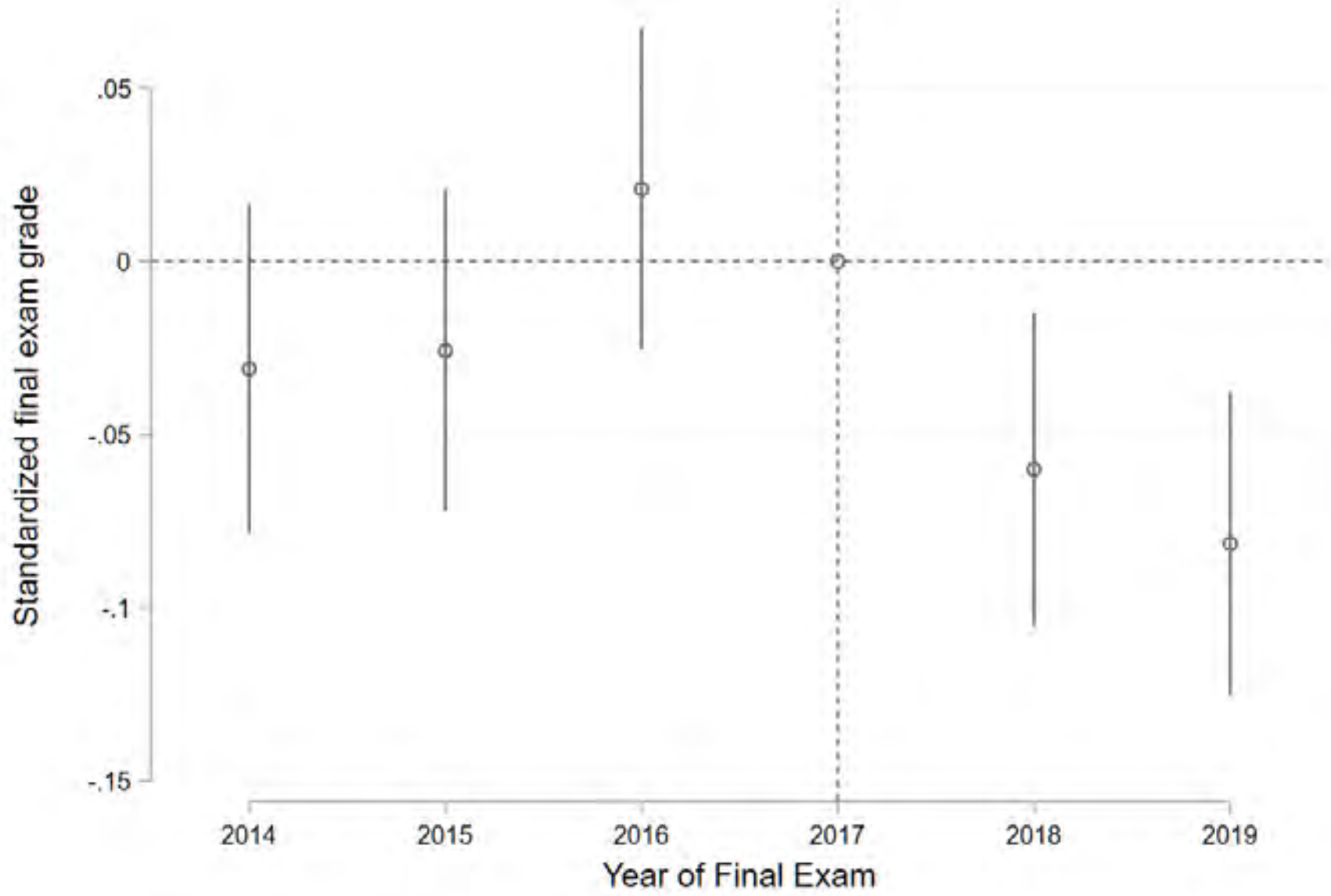


**B:** Probability of passing

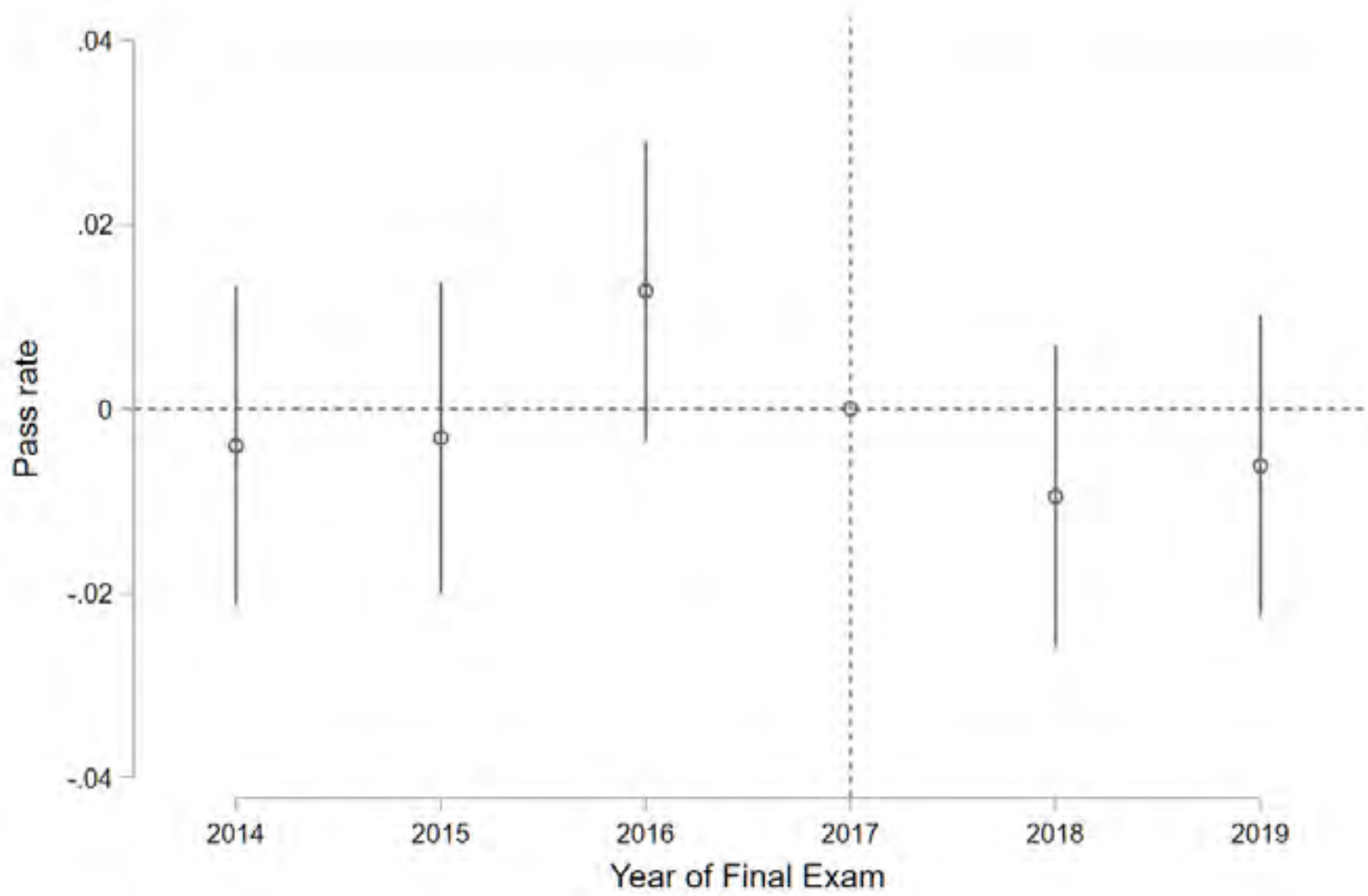


Notes: The panels plot event–study coefficients ($\beta_t$) with 99% confidence intervals from equation (1). Treated students are those with a Moroccan migration background. Exams in 2014–2017 occurred outside Ramadan, while those in 2018–2019 overlapped with Ramadan. Final-exam grade is standardized within track and exam year; the pass rate equals one if the student graduates (including after retakes). All specifications include the full set of controls, school–subtrack fixed effects, and exam-year fixed effects. Standard errors are clustered at the school–track–subtrack–year level. The horizontal dashed line marks zero effect.

**Figure A11:** Ramadan effects using continuous predicted fasting probability

**A:** Standardized final-exam grade

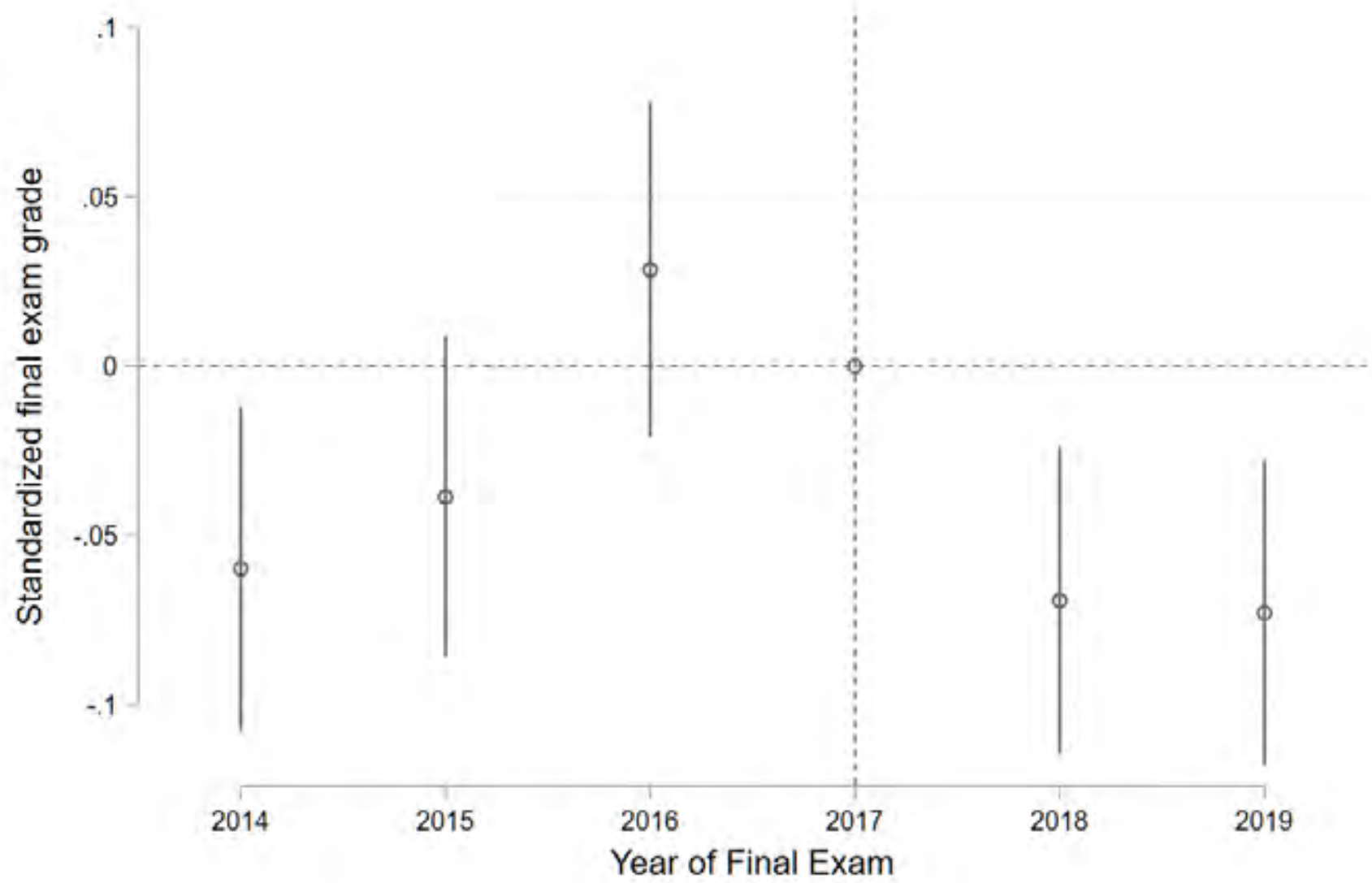


**B:** Probability of passing

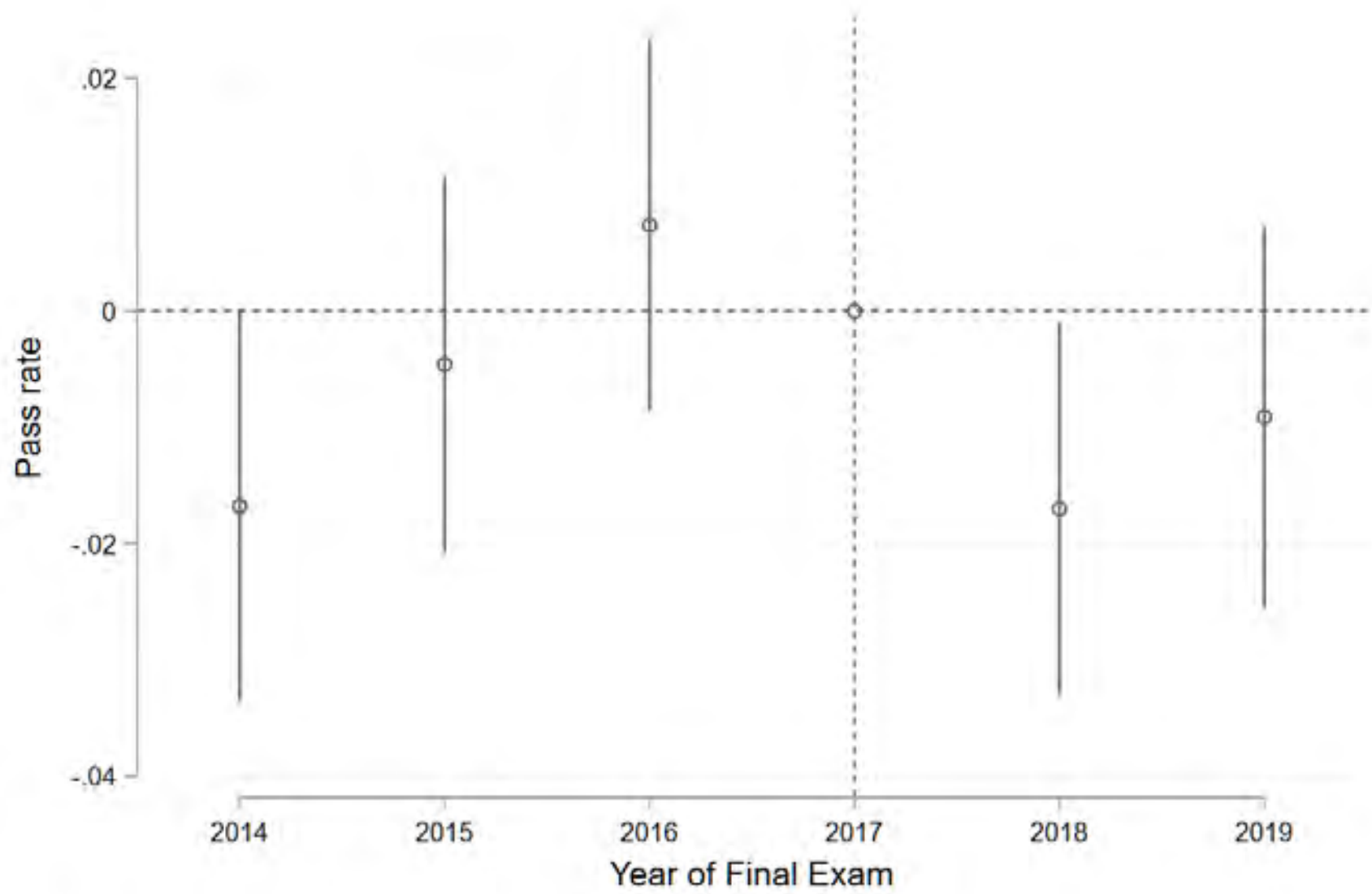


Notes: The panels plot event–study coefficients ($\beta_t$) with 99% confidence intervals from equation (1). Treatment intensity is measured continuously as the predicted fasting probability from our behavioural ML model (based on Moroccan- or Turkish-origin students). Outcome measures and controls match Figure A10. Standard errors are clustered at the school–track–subtrack–year level. The dashed line marks zero effect.

**Figure A12:** Ramadan exam impact across alternative fasting–probability thresholds

**A:** Standardized final exam grade    **B:** Pass rate

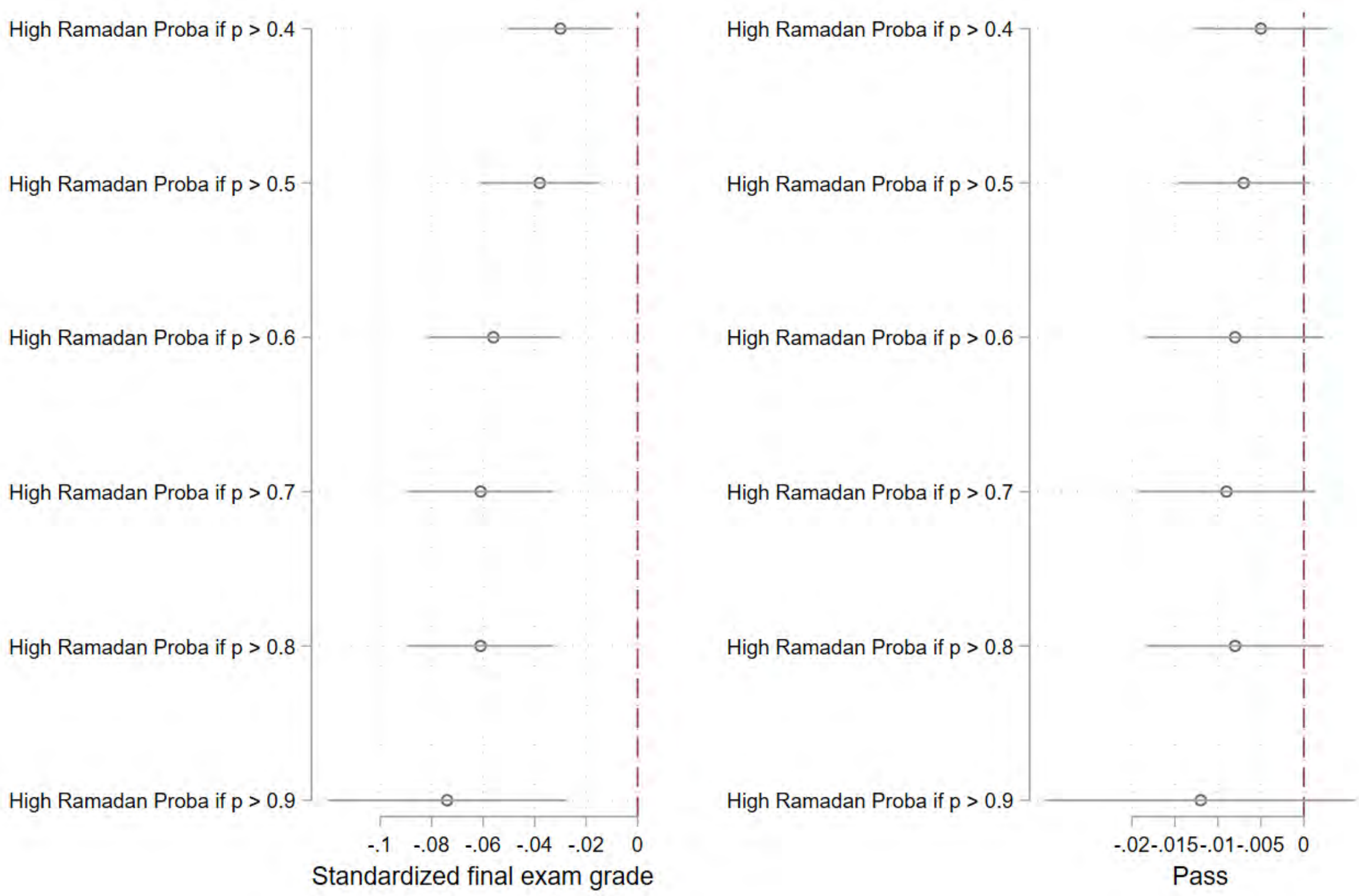


Notes: The figure reports difference-in-differences estimates from equation (2) with 99% confidence intervals. Treated students have a Moroccan or Turkish migration background and a predicted fasting probability $\geq$ the threshold indicated on the vertical axis (based on the penalized-logit model described in Section 4.3). Thresholds range from 0.40 to 0.90, providing a treatment-intensity validation. The dependent variables are: (A) standardized central-exam grade (mean 0, SD 1 within track × year); and (B) pass indicator (1 if graduated, incl. retakes). Exams in 2014–2017 did not overlap with Ramadan; exams in 2018–2019 did. All models include the full control set from Table B6, school–subtrack fixed effects, and exam-year fixed effects. Standard errors clustered at the school–track–subtrack–year level. The horizontal dashed line indicates zero effect. The increasing magnitude of estimates at higher thresholds is consistent with stronger treatment intensity among students most likely to fast.

**Figure A13:** Variation in Ramadan compliance probability across and within schools

**A:** Probability in schools and cohorts **B:** Difference in probability within schools

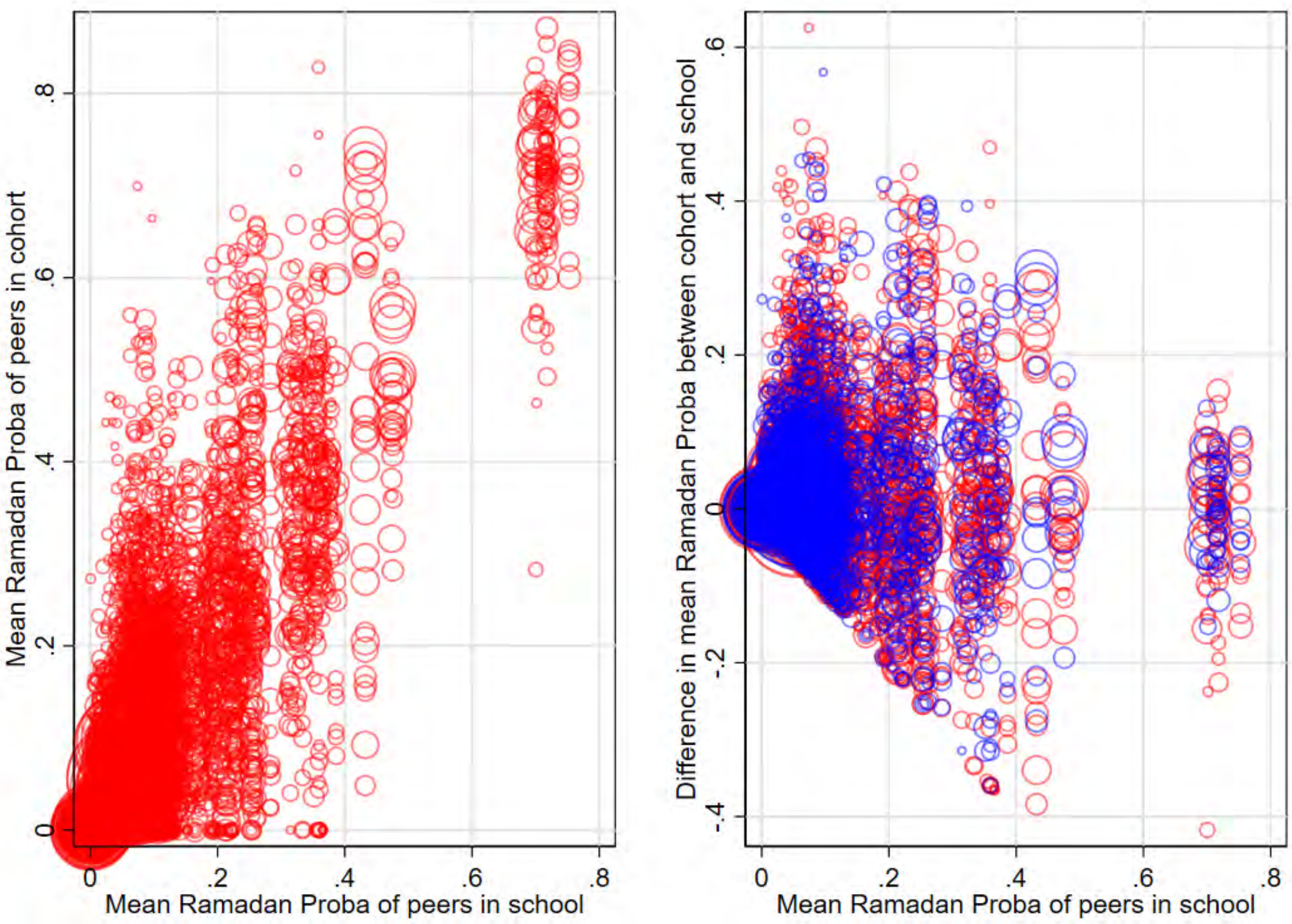


Notes: The left figure plots the mean Ramadan compliance probability in a student's school on the x-axis (leave-one-out-mean in same school) and the mean probability in a student's cohort on the y-axis (leave-one-out-mean in same subtrack-track-school-exam year) for the years 2014 to 2019. The size of the circles is determined by size of cohort. The right figure plots the (leave-one-out) mean continuous Ramadan probability of peers in school (leave-one-out-mean in same school) on the x-axis and the difference between the (leave-one-out-mean) continuous Ramadan probability in the cohort and in the school on the y-axis. The size of the circles is determined by size of cohort. The red circles define the cohorts that took their final exams in 2014-2017, while the blue circles define the cohorts that took their final exams in 2018-2019 (when Ramadan overlapped with the final exams).

**Figure A14:** Sensitivity of Ramadan effects to deviations from parallel trends (HonestDiD, main specification)

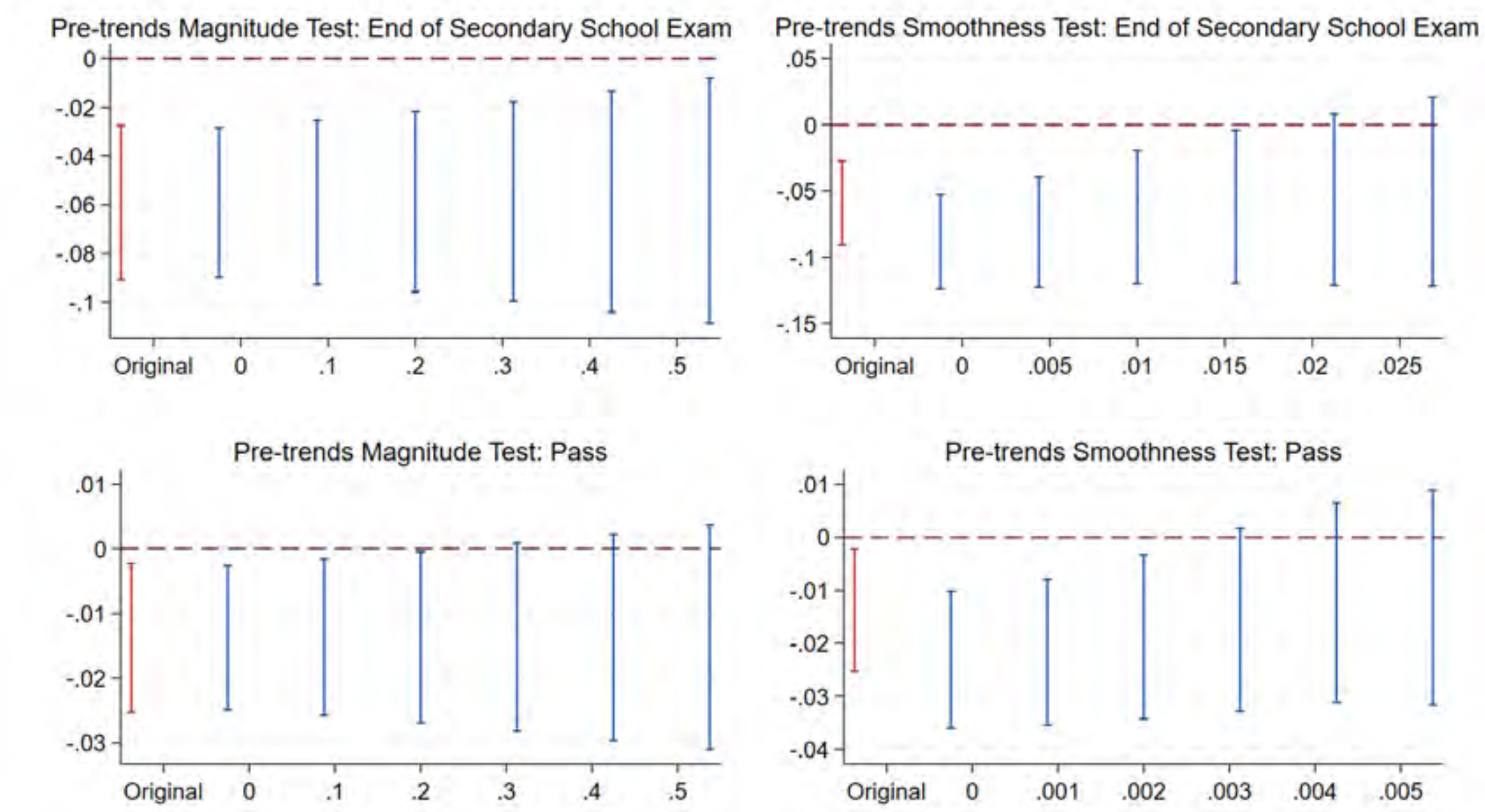


Notes: We implement the Rambachan and Roth (2023) sensitivity framework using the `honestdid` package for the high Ramadan-compliance specification. Red bars report the baseline post-Ramadan treatment effects. Blue intervals show the most negative or positive effects that remain consistent with bounded violations of parallel trends in levels (magnitude test) or slopes (smoothness test) calibrated from pre-treatment coefficients. The estimated Ramadan impact on exam performance remains negative and economically meaningful for all reasonably sized deviations, and would be eliminated only under implausibly large pre-trend departures relative to those observed before the overlap.

**Figure A15:** Heterogeneity in impact of Ramadan on secondary education outcomes by Ramadan probability of peers

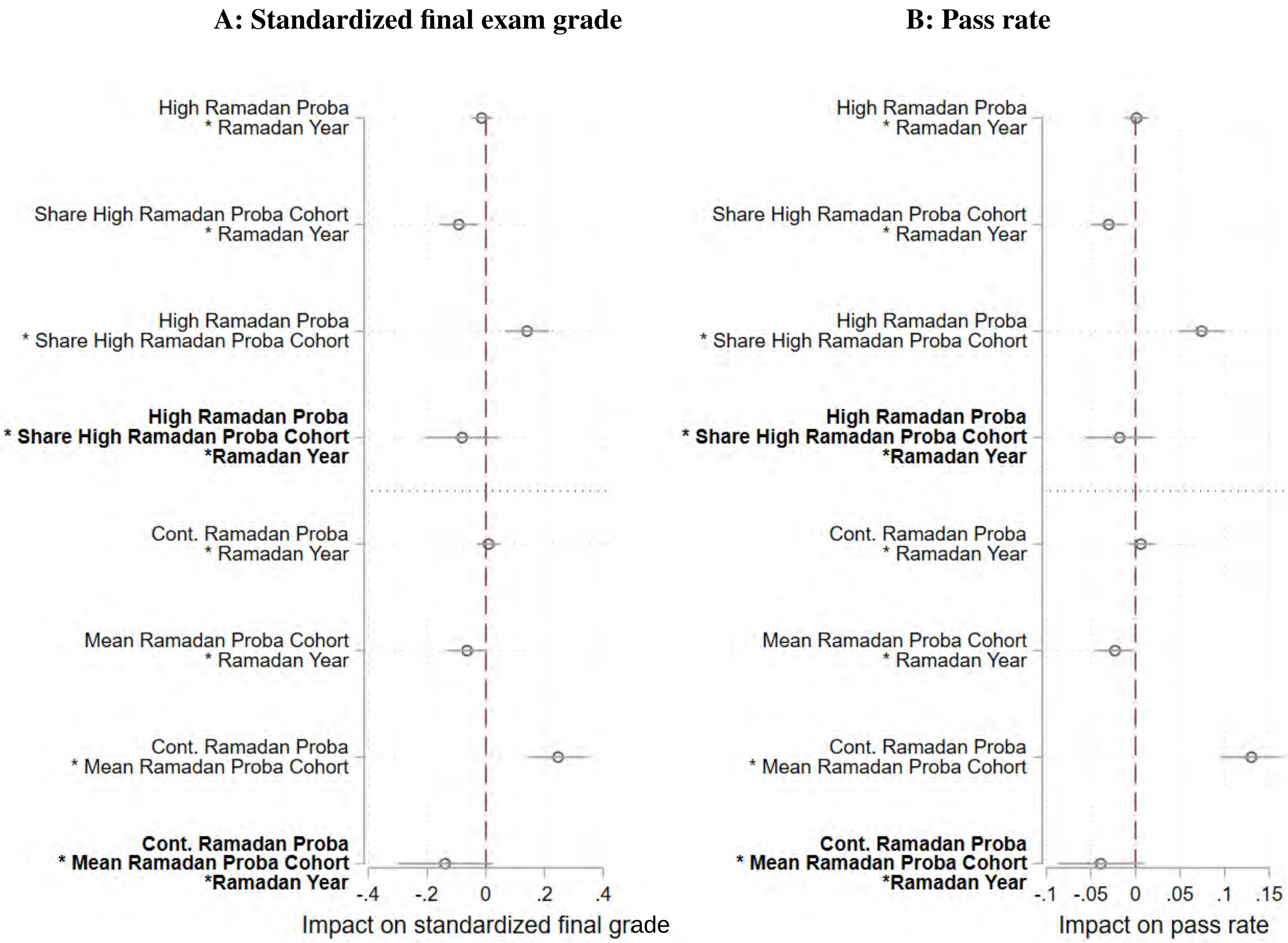


Notes: The figure plots triple-difference estimates with 99% confidence intervals from an extension of equation (2). Panel A (left) reports effects on standardized final-exam grades; Panel B (right) reports effects on the probability of passing. In the top row, treated students are those with a Moroccan or Turkish migration background who have a high predicted probability of fasting (*High Proba*). We interact the Ramadan treatment indicator with the cohort share of high-probability fasters (track × school × year). In the bottom row, treatment is defined continuously using predicted fasting probabilities for all students, interacted with the cohort-average probability. Bold y-axis labels denote the triple-difference coefficients (treatment × Ramadan-overlap years × peer fasting measure). All specifications include the full control set from Table B6, plus school–subtrack and exam-year fixed effects. Standard errors are clustered at the school–track–subtrack–year level. The horizontal dashed line marks zero effect.

**Figure A16:** Number of exam takers and exams by timing and migration background

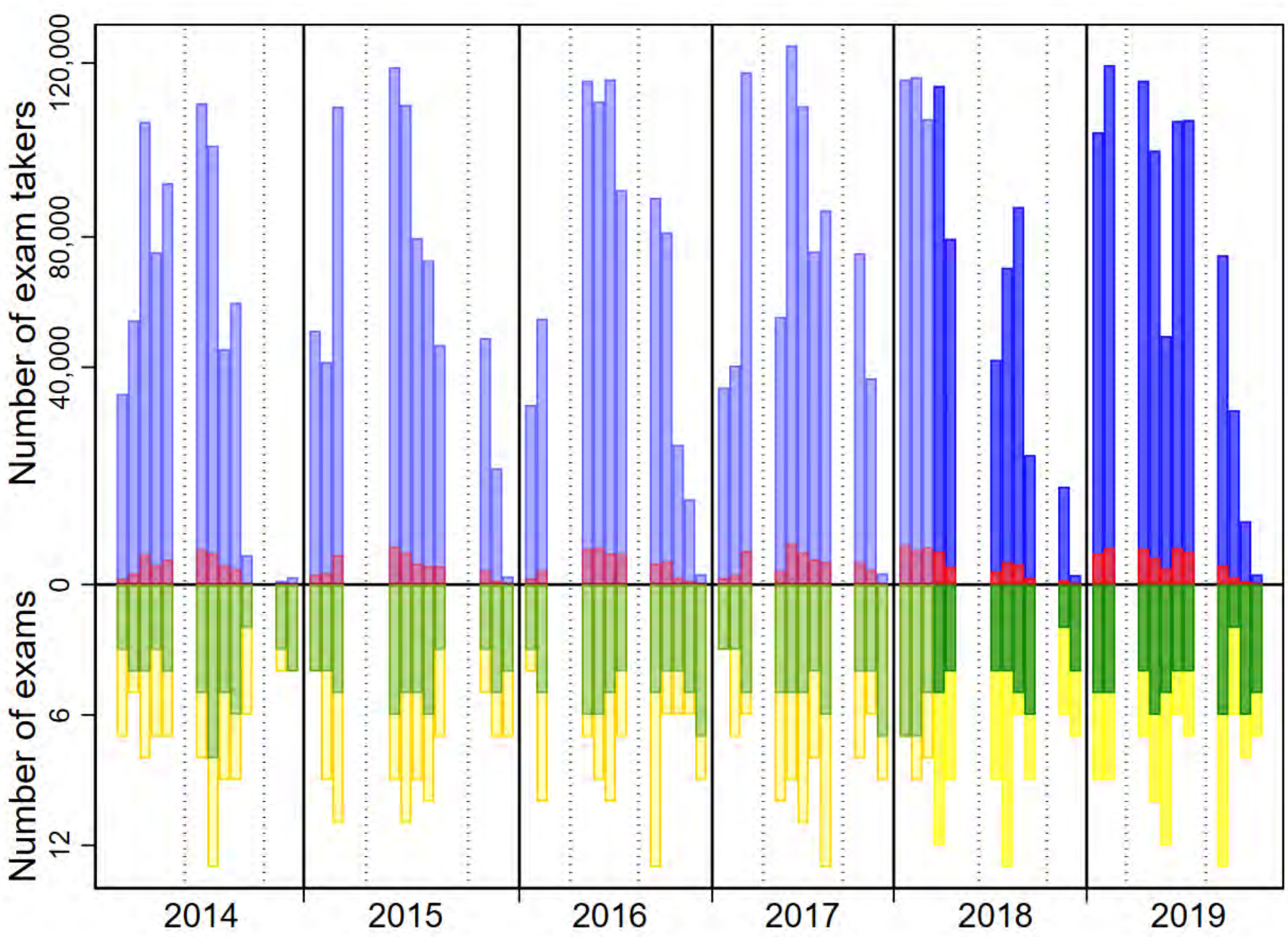


Notes: The figure plots on the upper panel the number of exam takers, with in red the number of students with a Moroccan or Turkish migration background and in blue the total number of exam takers on that day. In the bottom panel, it displays the number of exams per day, with in green the number of exams that take place in the afternoon and in yellow the number of exams that take place in the morning. The darker colors indicate exam days that took place during Ramadan.

**Figure A17:** Effect on final grade by Ramadan week

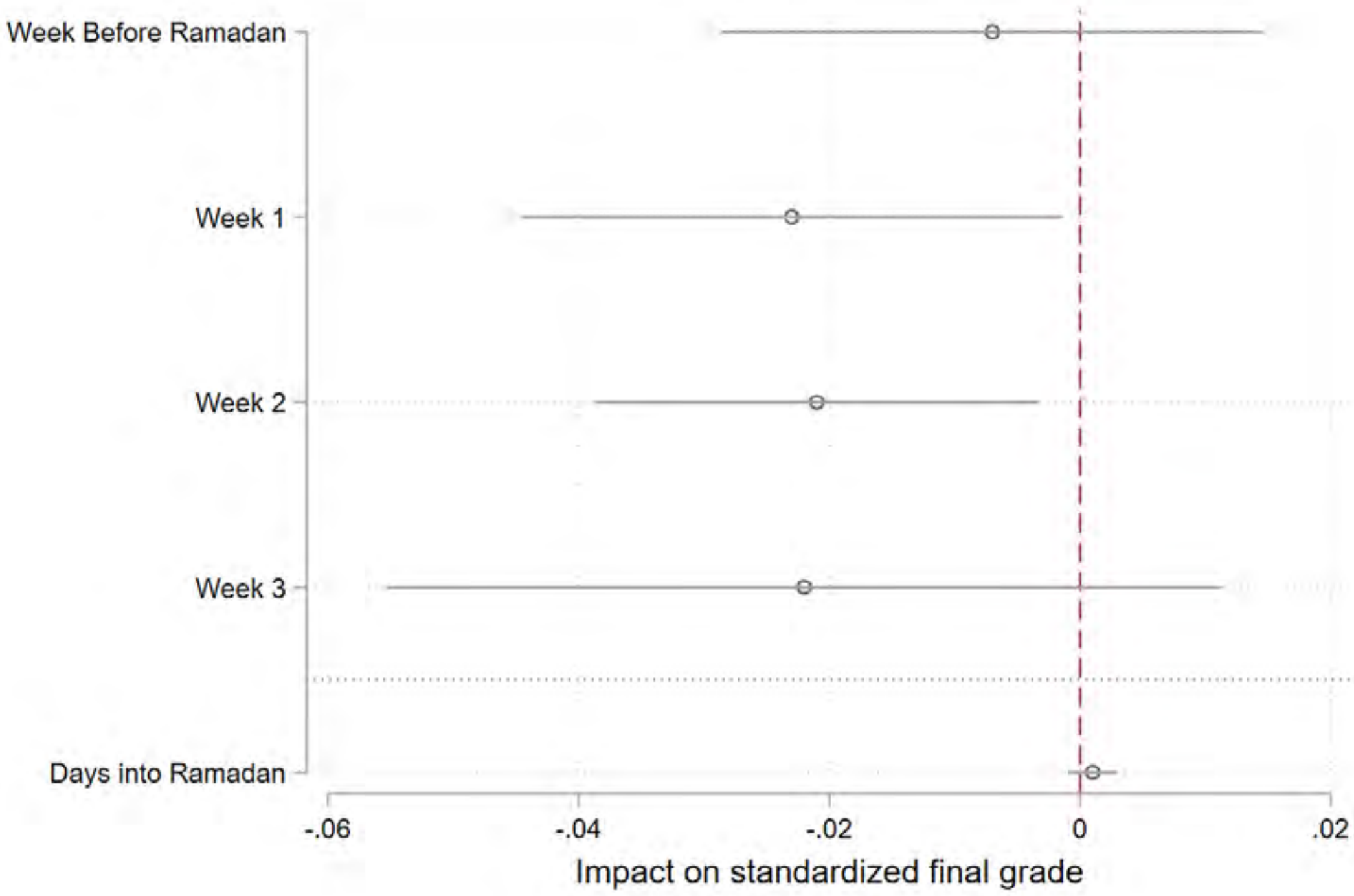


Notes: This figure plots the point estimates and 99% confidence intervals for the coefficients estimated using equations (C3) and (C4). Week 1, Week 2, and Week 3 measure the impact of Ramadan exposure during the respective week of Ramadan on the standardized central exam grade, using subject-level data. Week Before Ramadan refers to the week preceding Ramadan (observed only in 2018). Days into Ramadan captures the marginal effect of an additional day of Ramadan exposure ($\beta_2$ in equation (C4)). Treated students are those with a Moroccan or Turkish migration background and a high predicted Ramadan compliance probability. All controls from Table B6 are included, with school-track-exam subject and treatment type-exam subject-afternoon-two day exam fixed effects. Standard errors are clustered at the school-track-subject-year level. Results are shown for weeks 1 to 3 only, as no exams fell during the fourth week of Ramadan in either 2018 or 2019.

# B Additional tables

**Table B1:** Compliance with measures of Muslim religiosity by migration background

| | SIM Respondents | |
|---|---|---|
| | Moroccan Migration BkG | Turkish Migration BkG |
| | (1) | (2) |
| **Prays At Least Every Day** | 0.796 | 0.376 |
| | (.403) | (.484) |
| **Visits Mosque At Least Every Week** | 0.367 | 0.344 |
| | (.482) | (.475) |
| **Eats Halal Every Day** | 0.877 | 0.732 |
| | (.328) | (.443) |
| **Finds Religion Important** | 0.901 | 0.801 |
| | (.299) | (.399) |
| **Islam Rules** | 0.601 | 0.501 |
| **Should be Followed Completely** | (.490) | (.500) |
| Observations | 2,677 | 2,910 |

Notes: Self-reported compliance with measures of Muslim religiosity based on SIM (*Survey Integratie Minderheden*) data of respondents with a Moroccan migration background on the left and Turkish migration background on the right. Source: SIM data in 2006, 2011, 2015 and 2020. Observations are respondent-wave observations; respondents interviewed in more than one wave are counted once in the analysis sample of 5,400 respondents (see Appendix C.4).

**Table B2:** Comparison of SIM respondents and exam takers used in the analysis

| | Moroccan Background | | Turkish Background | |
|---|---|---|---|---|
| | SIM Sample | Exam Takers | SIM Sample | Exam Takers |
| | (1) | (2) | (3) | (4) |
| Age | 38.18 | 16.50 | 38.33 | 16.54 |
| | (15.32) | (0.98) | (15.23) | (0.98) |
| Share Below Age 45 in Neighbourhood | 0.615 | 0.608 | 0.609 | 0.597 |
| | (0.089) | (0.090) | (0.091) | (0.090) |
| Births per Capita (Neighbourhood) | 0.028 | 0.028 | 0.027 | 0.027 |
| | (0.007) | (0.008) | (0.008) | (0.009) |
| Average Household Size (Neighbourhood) | 2.107 | 2.165 | 2.145 | 2.188 |
| | (0.303) | (0.316) | (0.321) | (0.310) |
| Share Receiving Assistance Benefits | 0.059 | 0.058 | 0.058 | 0.056 |
| | (0.034) | (0.034) | (0.035) | (0.035) |
| Share Moroccan Background (Neighbourhood) | 0.107 | 0.114 | 0.076 | 0.074 |
| | (0.091) | (0.096) | (0.078) | (0.082) |
| Share Turkish Background (Neighbourhood) | 0.078 | 0.079 | 0.111 | 0.102 |
| | (0.075) | (0.073) | (0.092) | (0.085) |
| Distance to Moroccan Mosque (meters) | 2,068 | 1,975 | 3,394 | 3,989 |
| | (3,621) | (3,544) | (5,883) | (6,447) |
| Distance to Turkish Mosque (meters) | 2,204 | 2,271 | 1,485 | 1,742 |
| | (3,002) | (2,882) | (1,889) | (2,141) |
| Observations | 2,104 | 30,413 | 2,208 | 29,546 |

Notes: SIM = Survey Integratie Minderheden (2006–2020). Exam takers = all Moroccan/Turkish-origin students taking central exams (2014–2019). Neighbourhood-level variables are measured at 6-digit postcode level. Mosque distances are Euclidean distances to nearest mosque of matching ethnic denomination. The SIM sample is restricted to respondents that could be linked to neighbourhood-level data.

**Table B3:** Composition of treated group

| | **Students with** | |
|---|---|---|
| | Moroccan Migration BkG | Turkish Migration BkG |
| | (1) | (2) |
| **Moroc BkG** | 1.000 | 0.000 |
| **High Ramadan Proba** | 0.998 | 0.250 |
| **Continuous Ramadan Proba** | 0.883 | 0.565 |
| Observations | 30,413 | 29,546 |

Notes: Share of students in our sample by migration background that are ‘treated’ (compliant to the Ramadan fast) by treated specification.

**Table B4:** Ramadan effects on secondary school outcomes with stepwise inclusion of controls

| | Panel A: Final | | | | | | |
|---|---|---|---|---|---|---|---|
| | (1) | (2) | (3) | (4) | (5) | (6) | (7) |
| **Ramadan Exam Years** | - 0.007 | - 0.009 | - 0.016 | - 0.027 ** | - 0.047 *** | -0.045 *** | -0.058 *** |
| ***High Ramadan Proba** | (.015) | (.015) | (.013) | (.013) | (.010) | (.012) | (.010) |
| Mean exam grade | 6.1 | 6.1 | 6.1 | 6.1 | 6.1 | 6.1 | 6.1 |
| Share treated | 4.7% | 4.7% | 4.7% | 4.7% | 4.7% | 4.7% | 4.7% |
| Observations | 776,438 | 776,438 | 776,219 | 776,219 | 776,219 | 776,219 | 776,219 |
| | **Panel B: Pass** | | | | | | |
| | (1) | (2) | (3) | (4) | (5) | (6) | (7) |
| **Ramadan Exam Years** | - 0.011 ** | - 0.009 * | - 0.005 | - 0.007* | - 0.010*** | - 0.009** | - 0.011*** |
| ***High Ramadan Proba** | (.005) | (.005) | (.004) | (.004) | (.004) | (.004) | (.004) |
| Mean pass rate | 86.8% | 86.8% | 86.8% | 86.8% | 86.8% | 86.8% | 86.8% |
| Share treated | 4.7% | 4.7% | 4.7% | 4.7% | 4.7% | 4.7% | 4.7% |
| Observations | 776,504 | 776,504 | 776,285 | 776,285 | 776,284 | 776,285 | 776,284 |
| School-Subtrack f.e. | No | No | Yes | Yes | Yes | Yes | Yes |
| Exam year f.e. | No | Yes | Yes | Yes | Yes | Yes | Yes |
| Individual controls | No | No | No | Yes | Yes | Yes | Yes |
| Cont. exam control | No | No | No | No | Yes | No | Yes |
| CITO test control | No | No | No | No | No | Yes | Yes |

Notes: Estimates of $\beta$ from equation (2) for students with a high predicted probability of fasting. Panel A reports effects on the standardized final-exam grade; Panel B shows effects on the probability of graduating (including retakes). Columns add controls and fixed effects sequentially: exam-year FE, school–subtrack FE, individual characteristics, continuous-assessment grade, and CITO score. "Mean exam grade" and "Mean pass rate" refer to treated students in pre-overlap years (2014–2017). Standard errors clustered at the school–track–subtrack–year level. $^{*}p < 0.10$, ** $p < 0.05$, *** $p < 0.01$.

**Table B5:** Impact of Ramadan on education outcomes of students, standard errors clustered at school-exam year level

| | Secondary School Outcomes | | | | | |
|---|---|---|---|---|---|---|
| | **High Ramadan Proba** | | **Moroc BkG** | | **Cont. Ramadan Proba** | |
| | Final | Pass | Final | Pass | Final | Pass |
| | (1) | (2) | (3) | (4) | (5) | (6) |
| **Ramadan Exam Years** | - 0.058*** | - 0.011*** | - 0.059*** | - 0.009** | - 0.052*** | - 0.011** |
| ***Treated** | (.012) | (.004) | (.013) | (.004) | (.014) | (.004) |
| School-Subtrack f.e. | Yes | Yes | Yes | Yes | Yes | Yes |
| Exam year f.e. | Yes | Yes | Yes | Yes | Yes | Yes |
| All controls | Yes | Yes | Yes | Yes | Yes | Yes |
| Mean outcome before | 6.10 | 86.8% | 6.13 | 87.4% | 6.08 | 86.0% |
| Share treated | 4.7% | 4.7% | 3.9% | 3.9% | 5.5% | 5.5% |
| Observations | 776,219 | 776,284 | 746,697 | 746,742 | 776,219 | 776,284 |

Notes: The table shows point estimates for the coefficient $\beta$ estimated using equation (2) for the 3 different treatment group specifications. Final is the average of the central exam grades standardized with mean zero and standard deviation one by track and final exam year. Pass is a dummy indicating that a student passed the final exams (including retakes) and graduated from secondary education. The difference in number of observations between the analyses with Final and Pass as outcomes can be explained by students not taking the exam during the first period, but during the first or second retake due to e.g. illness. Mean outcome before is the average outcome for treated students in the pre-treatment years 2014 to 2017. Controls included are: gender, age at exam, end of primary school test score (standardized by year), continuous exam grade (standardized by track and exam year), moved up from lower track or failed the final exams last year, number of siblings, a dummy for being Moroccan or Turkish, time spent abroad, parental income, unemployment or illness benefits, and on the neighbourhood level: the share receiving assistance benefits, the average household size, the ratio under 45 years old, fertility rate, share with Turkish migration background, share with Moroccan migration background. Robust standard errors are clustered at the school-exam year level in parentheses. $^{*}p < 0.10$, ** $p < 0.05$, *** $p < 0.01$.

**Table B6:** Ramadan effects under alternative treatment definitions

| | High Ramadan Proba. | | Moroccan BkG | | Continuous Ramadan Proba. | |
|---|---|---|---|---|---|---|
| | Final | Pass | Final | Pass | Final | Pass |
| | (1) | (2) | (3) | (4) | (5) | (6) |
| **Ramadan Exam Years** | -0.058*** | -0.011*** | -0.059*** | -0.009** | -0.052*** | -0.011** |
| *** Treated** | (0.010) | (0.004) | (0.011) | (0.004) | (0.011) | (0.004) |
| School–subtrack FE | Yes | Yes | Yes | Yes | Yes | Yes |
| Exam-year FE | Yes | Yes | Yes | Yes | Yes | Yes |
| All controls | Yes | Yes | Yes | Yes | Yes | Yes |
| Mean outcome before | 6.10 | 86.8% | 6.13 | 87.4% | 6.08 | 86.0% |
| Share treated | 4.7% | 4.7% | 3.9% | 3.9% | 5.5% | 5.5% |
| Observations | 776,219 | 776,284 | 746,697 | 746,742 | 776,219 | 776,284 |

Notes: The table reports estimates of $\beta$ from equation (2) using three treatment definitions: (i) high predicted probability of fasting, (ii) Moroccan migration background, and (iii) the continuous fasting-probability measure. *Final* is the standardized central-exam grade; *Pass* indicates graduation (including retakes). Differences in observations between Final and Pass arise because some students only sat retake exams. "Mean outcome before" refers to treated students in pre-Ramadan-overlap years (2014–2017). All specifications include student demographics, prior achievement, family composition, migration-background indicators, household income and benefits, and neighbourhood characteristics. School–subtrack and exam-year fixed effects are included. Standard errors clustered at the school–track–subtrack–year level. $^{*}p < 0.10$, ** $p < 0.05$, *** $p < 0.01$.

**Table B7:** Balance table student characteristics before and after Ramadan

| | High Ramadan probability | | | Moroccan migration background | | |
|---|---|---|---|---|---|---|
| | No | Yes | diff-in -diff | No | Yes | diff-in -diff |
| | (1) | (2) | (3) | (4) | (5) | (6) |
| **Family characteristics** | | | | | | |
| Income decile | 5.778 | 2.617 | -0.029 | 5.857 | 2.562 | -0.022 |
| | (2.787) | (1.951) | (0.026) | (2.767) | (1.959) | (0.029) |
| Unemployment benefits | 0.119 | 0.123 | -0.002 | 0.117 | 0.114 | -0.001 |
| | (0.324) | (0.328) | (0.003) | (0.322) | (0.318) | (0.004) |
| Illness benefits | 0.090 | 0.279 | -0.001 | 0.083 | 0.279 | 0.005 |
| | (0.286) | (0.448) | (0.005) | (0.276) | (0.449) | (0.005) |
| **Student characteristics** | | | | | | |
| Male | 0.495 | 0.455 | 0.005 | 0.494 | 0.475 | 0.003 |
| | (0.500) | (0.498) | (0.005) | (0.500) | (0.499) | (0.006) |
| Age at exam | 16.67 | 16.51 | -0.001 | 16.67 | 16.50 | 0.003 |
| | (1.022) | (0.967) | (0.008) | (1.024) | (0.970) | (0.008) |
| moved up | 0.039 | 0.041 | -0.002 | 0.039 | 0.044 | -0.003 |
| from lower track | (0.193) | (0.198) | (0.002) | (0.193) | (0.205) | (0.002) |
| Failed exam | 0.044 | 0.066 | 0.003 | 0.043 | 0.062 | 0.003 |
| year before | (0.205) | (0.248) | (0.003) | (0.202) | (0.240) | (0.003) |
| Stand. primary school | 0.038 | -0.590 | 0.039*** | 0.057 | -0.559 | 0.033*** |
| test | (0.956) | (0.979) | (0.007) | (0.950) | (0.978) | (0.008) |
| Stand. cont. exam | 0.028 | -0.355 | 0.019 | 0.039 | -0.341 | 0.011 |
| grade | (0.999) | (0.952) | (0.012) | (0.998) | (0.963) | (0.013) |
| Siblings | 2.209 | 3.198 | -0.065*** | 2.208 | 3.202 | -0.075*** |
| | (0.929) | (1.218) | (0.013) | (0.932) | (1.275) | (0.015) |
| Time spent abroad | 0.082 | 0.134 | -0.041*** | 0.081 | 0.113 | -0.035*** |
| | (0.752) | (0.869) | (0.009) | (0.748) | (0.782) | (0.009) |
| **Neighbourhood characteristics** | | | | | | |
| Share assistance | 0.020 | 0.064 | -0.002*** | 0.020 | 0.059 | -0.002*** |
| benefits | (0.023) | (0.036) | (0.000) | (0.022) | (0.035) | (0.000) |
| Average household | 2.381 | 2.156 | -0.010** | 2.387 | 2.159 | -0.007 |
| size | (0.448) | (0.298) | (0.004) | (0.450) | (0.313) | (0.004) |
| Share under 45 | 0.523 | 0.622 | 0.005*** | 0.522 | 0.611 | 0.006*** |
| years old | (0.089) | (0.088) | (0.001) | (0.088) | (0.090) | (0.001) |
| Fertility rate | 0.023 | 0.028 | -0.002*** | 0.023 | 0.028 | -0.002*** |
| | (0.011) | (0.028) | (0.000) | (0.011) | (0.008) | (0.000) |
| Share Turkish mig bkg | 0.017 | 0.097 | -0.001 | 0.015 | 0.080 | 0.000 |
| | (0.035) | (0.083) | (0.001) | (0.030) | (0.074) | (0.001) |
| Share Moroccan mig bkg | 0.015 | 0.123 | 0.003*** | 0.014 | 0.115 | 0.001 |
| | (0.031) | (0.098) | (0.001) | (0.029) | (0.096) | (0.001) |
| School-Subtrack f.e. | | | Yes | | | Yes |
| Exam year f.e. | | | Yes | | | Yes |
| All controls | | | Yes | | | Yes |
| Share treated | | | 0.052 | | | 0.045 |

Notes: This table shows the sample means (standard deviations in parentheses) split by control and treatment group for each treatment definition: high Ramadan probability in Col (1)-(3) and Moroccan migration background in Col. (4)-(6). Columns (3) and (6) report the estimated coefficient $\beta$ from Equation (2) with $y_{i,p,s,t}$ being the variables listed in the rows. All control variables other than the characteristic taken as outcome in that specification are included, as in the main specification. Robust standard errors clustered at the school-track-subtrack exam year level in parentheses. $^{*}p < 0.10$, ** $p < 0.05$, *** $p < 0.01$.

**Table B8:** Impact of Ramadan on secondary school outcomes controlling for sibling and family fixed effects

| | Secondary School Outcomes | | | |
|---|---|---|---|---|
| | **Incl. Sibling f.e.** | | **Incl. Family f.e.** | |
| | Final | Pass | Final | Pass |
| | (1) | (2) | (3) | (4) |
| **Ramadan Exam Years** | - 0.059*** | - 0.014** | - 0.068*** | - 0.018*** |
| *** High Ramadan Proba** | (.014) | (.007) | (.013) | (.005) |
| School-Subtrack f.e. | Yes | Yes | Yes | Yes |
| Exam year f.e. | Yes | Yes | Yes | Yes |
| Sibling f.e. | Yes | Yes | No | No |
| Family f.e. | No | No | Yes | Yes |
| All controls | Yes | Yes | Yes | Yes |
| Mean outcome before | 6.03 | 81.8% | 6.07 | 84.9% |
| Share treated | 4.6% | 4.6% | 5.4% | 5.4% |
| Observations | 408,017 | 408,060 | 493,477 | 493,528 |

Notes: The table shows point estimates for the coefficient $\beta$ estimated using equation (2). Treated students in this table are students with a Moroccan or Turkish migration background that have a high Ramadan probability, as predicted by our machine learning model. In Column 1 and 2 we include sibling fixed effects, in Column 3 and 4 family fixed effects (comparing students not only to their siblings but also to their cousins). Final is the average of the central exam grades standardized with mean zero and standard deviation one by track and final exam year. Pass is a dummy indicating that a student passed the final exams and graduated from secondary education. Mean outcome before is the average outcome for treated students in the pre-treatment years 2014 to 2017. All controls are included, as in Table B6. Robust standard errors are clustered at the school-track-subtrack-year level in parentheses. $^{*}p < 0.10$, ** $p < 0.05$, *** $p < 0.01$.

**Table B9:** Placebo impact of Ramadan on secondary school outcomes

| | **Secondary School Outcomes** | |
|---|---|---|
| | Final | Pass |
| | (1) | (2) |
| *Panel A: Ramadan Probability Based on Within-Turk ML Model* | | |
| **Ramadan Exam Years** | - 0.001 | - 0.000 |
| ***High Ramadan Proba** | (.008) | (.003) |
| Mean exam grade | 6.02 | 84.7% |
| Share treated | 4.9% | 4.9% |
| Observations | 707,501 | 707,561 |
| School-Subtrack f.e. | Yes | Yes |
| Exam year f.e. | Yes | Yes |
| All controls | Yes | Yes |
| *Panel B: Ramadan Probability Based on Within-Moroc ML Model* | | |
| **Ramadan Exam Years** | 0.013 | - 0.000 |
| ***High Ramadan Proba** | (.008) | (.003) |
| Mean exam grade | 6.13 | 87.4% |
| Share treated | 4.9% | 4.9% |
| Observations | 707,501 | 707,561 |
| School-Subtrack f.e. | Yes | Yes |
| Exam year f.e. | Yes | Yes |
| All controls | Yes | Yes |

Notes: The table shows point estimates for the coefficient $\beta$ estimated using equation (2). In Panel A, 'treated' students are defined as students with a non-Muslim migration background or without a migration background that are classified as high Ramadan probability according to predictions made by our within-Turk ML model. In Panel B, 'treated' students are defined as students with a non-Muslim migration background or without a migration background that are classified as high Ramadan probability according to predictions made by our within-Moroccan ML model. Final is the average of the central exam grades standardized with mean zero and standard deviation one by track and final exam year. Pass is a dummy indicating that a student passed the final exams and graduated from secondary education. Mean outcome before is the average outcome for treated students in the pre-treatment years 2014 to 2017. All controls are included, as in Table 1. Robust standard errors are clustered at the school-track-subtrack-year level in parentheses. $^{*}p < 0.10$, ** $p < 0.05$, *** $p < 0.01$.

**Table B10:** Impact of Ramadan at subject level

| | Secondary School Outcomes | | |
|---|---|---|---|
| | **High Ramadan Proba** | **Moroc BkG** | **Cont. Ramadan Proba** |
| | Final | Final | Final |
| | (1) | (2) | (3) |
| **Ramadan Exam Years** | - 0.032*** | - 0.037*** | - 0.028*** |
| ***Treated** | (.006) | (.006) | (.007) |
| School-Track-Subject f.e. | Yes | Yes | Yes |
| Exam year f.e. | Yes | Yes | Yes |
| Treated-Subject-Afternoon-Two day f.e. | Yes | Yes | Yes |
| All controls | Yes | Yes | Yes |
| Mean outcome before | 6.13 | 6.17 | 6.11 |
| Share treated | 4.13% | 3.40% | 4.83% |
| Observations | 4,731,843 | 4,573,531 | 4,731,846 |

Notes: The table shows point estimates for the coefficient $\beta$ estimated using equation (C1) for the 3 different treatment group specifications, based on the subject level data. The dependent variable is the grade obtained in the exam (after retakes) standardized at subject-track-year level. All controls are included, as in Table B6. Mean outcome before is the average outcome for treated students in the pre-treatment years 2014 to 2017. Robust standard errors are clustered at the school-track-subject-year level in parentheses. $^{*}p < 0.10$, ** $p < 0.05$, *** $p < 0.01$.

**Table B11:** DiD estimates by type of subject using high Ramadan probability as treated

| | Secondary School Outcomes | | |
|---|---|---|---|
| | Memory-based | Languages | Math |
| | Final | Final | Final |
| | (1) | (2) | (3) |
| **Ramadan Exam Years** | - 0.042*** | - 0.024*** | - 0.089*** |
| *** High Ramadan Proba** | (.014) | (.010) | (.025) |
| School-Subtrack f.e. | Yes | Yes | Yes |
| Exam year f.e. | Yes | Yes | Yes |
| All controls | Yes | Yes | Yes |
| Mean exam grade | 6.04 | 6.19 | 6.21 |
| Share treated | 4.1% | 4.5% | 4.2% |
| Observations | 867,603 | 1,930,649 | 702,153 |
| No. students | 583,126 | 729,274 | 659,989 |

Notes: The table shows point estimates for the coefficient $\beta$ estimated using equation (C1), for three groups of exams: languages (reading comprehension tests in Dutch, English, German and French), more memory-based exams (history, geography and biology), and math. The dependent variable is the grade obtained in an exam (after retakes) standardized at subject-track-year level. We include school-track-exam subject and treatment type-exam subject-afternoon-two day exam fixed effects. Robust standard errors are clustered at the school-track-subject-year level in parentheses. All controls are included, as in Table B6. $^{*}p < 0.10$, ** $p < 0.05$, *** $p < 0.01$.

**Table B12:** Impact of Ramadan on education outcomes of students with a high Ramadan probability

| | **Secondary School Outcomes**<br>Final grade<br>(1) |
|---|---|
| **DiD** | - 0.040*** |
| | (.014) |
| **DiD *Afternoon** | 0.010 |
| | (.015) |
| School-Track-Subject f.e. | Yes |
| Treated-Subject-Afternoon-Two day f.e. | Yes |
| Exam year f.e. | Yes |
| All controls | Yes |
| Mean outcome before | 6.13 |
| Share treated | 4.13% |
| Observations | 4,731,843 |

Notes: The table shows point estimates for the coefficient $\beta_1$ (DiD) and for the coefficient $\beta_4$ on the interaction between the DiD term and a dummy that takes value 1 if the exam took place in the afternoon, estimated using equation (C2), based on the subject level data. Treated students in this table are students with a Moroccan or Turkish migration background that have a high Ramadan probability, as predicted by our machine learning model. The dependent variable is the grade obtained in an exam (after retakes) standardized at subject-track-year level. Mean outcome before is the average outcome for treated students in the pre-treatment years 2014 to 2017. All controls are included, as in Table B6. Robust standard errors are clustered at the school-track-subject-year level in parentheses. $^{*}p < 0.10$, ** $p < 0.05$, *** $p < 0.01$.

**Table B13:** Ramadan impact by size of *extended* family networks

| | **Secondary School Outcomes** | | | |
|---|---|---|---|---|
| | Small extended family<br>Final<br>(1) | Large extended family<br>Final<br>(2) | Small extended family<br>Pass<br>(3) | Large extended family<br>Pass<br>(4) |
| **Ramadan Exam Years** | -0.054*** | -0.065*** | -0.010** | -0.012** |
| *** High Ramadan Proba** | (0.013) | (0.013) | (0.005) | (0.005) |
| School–Subtrack FE | Yes | Yes | Yes | Yes |
| Exam year FE | Yes | Yes | Yes | Yes |
| Mean exam grade (treated) | 6.12 | 6.07 | 87.2% | 86.3% |
| Share treated | 4.5% | 5.0% | 4.5% | 5.0% |
| All controls | Yes | Yes | Yes | Yes |
| Observations | 423,221 | 352,488 | 423,261 | 352,513 |

Notes: The table shows DiD estimates from equation (2). Treated students are those with a Moroccan or Turkish migration background who have a high predicted probability of fasting. We split the sample by *extended family size living in the Netherlands*—below vs. above the median number of linked relatives (siblings, parents, grandparents, aunts, uncles, cousins). This measure is distinct from the immediate household-size information used in training the SCP/SIM–based fasting-probability model. Standard errors clustered at the school–track–subtrack–year level. $^{*}p < 0.10$, $^{**}p < 0.05$, $^{***}p < 0.01$.

**Table B14:** Impact of Ramadan on Longer-Term Outcomes

| | Longer Term Outcomes | | |
|---|---|---|---|
| | Grade retention | Adult education | Drop out |
| | (1) | (2) | (3) |
| **Ramadan Exam Years** | 0.007*** | 0.004 | 0.004 |
| ***High Ramadan Proba** | (.003) | (.002) | (.004) |
| School-Subtrack f.e. | Yes | Yes | Yes |
| Exam year f.e. | Yes | Yes | Yes |
| All controls | Yes | Yes | Yes |
| Share treated | 4.70% | 4.70% | 4.70% |
| Mean outcome | 5.60% | 4.94% | 5.01% |
| Observations | 776,284 | 776,284 | 776,284 |

Notes: The table shows point estimates for the coefficient $\beta$ estimated using equation (2). Treated students in this figure are students with a Moroccan or Turkish migration background that have a high Ramadan probability, as predicted by our machine learning model. Grade retention is a dummy that takes value one if the student repeats the final year in the year after the final exam. Adult education is a dummy that takes value one if the student goes into adult education in the year after the final exam. Drop out is a dummy that takes value one if the student is not registered for any education in the year after the final exam. Mean outcome before is the average outcome for treated students in the pre-treatment years 2014 to 2017. All controls are included, as in Table B6. Robust standard errors are clustered at the school-track-subtrack-year level in parentheses. $^{*}p < 0.10$, ** $p < 0.05$, *** $p < 0.01$.

# C Data Appendix

## C.1 Construction of sample

We use administrative data from Statistics Netherlands which contains information on all individuals who are registered in a municipality since 1995.[1] We start with the registry of exam takers (*EXAMVOTAB*), select all exam takers between 2014 and 2019 (where exam year is the school year plus one, because students start the final school year in September of, i.e., 2013 and take the exam in May 2014) and drop entries for which the final grade is missing,[2] which gives us a sample of 983,837 individuals (of whom some take more than one exam, so we have a total of 1,048,400 observations). We match them to the track and sub-track they are in using the education reference book (*OPLEIDINGSNRREFV26*).

## C.2 Construction of variables

We match the exam takers in our sample to their demographic characteristics (country of birth, country of origin, country of birth of parents, gender, birth date and migration generation) in the registry of persons (*GBAPERSOONTAB*). The parent-child registry (*KINDOUDERTAB*) is used to link the exam takers in our sample to their parents such that we can determine outcomes for the parents. We match parents to their demographic characteristics (country of birth, country of origin, birth date and country of birth of parents) in the registry of persons. We also use parents to identify the rest of the (extended) family to use in a family fixed effects analysis. We first use the identifying number of the mother to identify an exam taker's siblings, then we use it to identify the grandparents. We use the grandparents to identify aunts and uncles and then link those aunts and uncles to their children to identify cousins. We also do this for the father to identify the father's side of the family. Due to the problem of overlapping families, the family fixed effects are based on the mother of the father or, if this is not available, the mother of the mother, or, if this is not available the mother (only siblings) or father (only siblings). Treated students suffer from more limitations than control students, because in order to identify the extended family we need to identify the grandparents, which are often not available in the

[1]The administrative data from Statistics Netherlands is available at a remote-access facility after signing a confidentiality agreement.

[2]The number of missing observations in the exam years 2016 and 2019 is unusually high, but these entries refer to students that were already pre-registered in the exam grade dataset in the year before the final year for policy reasons, and not to students in the final year taking the exams.

administrative data for students with a migration background. Therefore, while for students in our control group we can identify for 95% the extended family (and compare the exam taker not only with siblings but also with cousins), we can only do this for 77% of the students in our treatment group.

Country of origin is defined as the country of birth if a person is born abroad or, if born in the Netherlands, the country of birth of the mother, or, if the mother is also born in the Netherlands, the country of birth of the father. We construct a new variable that also considers third generation migrants, by replacing the country of origin with the country of origin of the mother if the country of origin of the exam taker is the Netherlands, or the country of origin of the father if the country of origin of the mother is the Netherlands. We classify exam takers as Muslim if this new country of origin is a Muslim majority country (see Table C1).[3]

We use birth date from the registry of persons to define the age at exam (using the 1st of May of the exam year). We add information from the registry with the information on the individual's obtained test score at the standardized test at the end of primary education (*CITOTAB*). This registry is only available from 2006 to 2019, so given that exam takers took the CITO test 4 to 6 years before their final exam we match the exam takers to the CITOTAB 2006-2019. We can match 75.4% of the exam takers to their obtained CITO test score. This leaves us with a sample of 841,376 observations (789,259 unique students).

We aggregate the new country of origin variable at the school level to create a variable with the proportion of Moroccans in each school and track. We then divide this variable in four quantiles.

We then match the parents to data on labour income (*SECMWERKNDGAMNDBEDRAGBUS*), unemployment benefits (*SECMWERKLMNDBEDRAGBUS*), income from profits (*SECMZLFMNDBEDRAGBUS*), and illness benefits (*SECMZIEKTEAOMNDBEDRAGBUS*). We aggregate the income data to yearly income data and only keep data on income in the year before the exam was taken. We generate a dummy variable that equals one if one of the parents receives unemployment benefits and do the same for illness benefits. We sum all sources of income to create a variable on total parental income and divide this in year-specific deciles.

In our analysis we make use of different spatial data: we exploit several characteristics of the students' neighbourhood of residence and we compute (approximate) distances between the students' residences and the closest mosque and primary school. In order to link students

[3]We exclude Indonesia, even though it is a Muslim-majority country, because migrants from Indonesia to the Netherlands are a highly selected and mostly non-Muslim group.

**Table C1:** Muslim majority countries

| Country | Share Muslims (in %) |
|---|---|
| Morocco | 99 |
| Somalia | 100 |
| Afghanistan | 100 |
| Tunisia | 99 |
| Iran | 99 |
| Mauritania | 100 |
| Yemen | 99 |
| Iraq | 97 |
| Maldives | 100 |
| Comoros | 98 |
| Niger | 99 |
| Turkey | 100 |
| Algeria | 99 |
| Azerbaijan | 97 |
| Jordan | 97 |
| Uzbekistan | 88 |
| Djibouti | 94 |
| Libya | 97 |
| Senegal | 96 |
| Pakistan | 97 |
| Tajikistan | 98 |
| Gambia | 96 |
| Egypt | 90 |
| Mali | 94 |
| Turkmenistan | 93 |
| Syria | 87 |
| Saudi Arabia | 99 |
| Bangladesh | 89 |
| Sudan | 100 |
| Kyrgyzstan | 90 |
| Indonesia | 87 |
| Oman | 86 |
| Guinea | 89 |
| Albania | 57 |
| Sierra Leone | 77 |
| Brunei | 79 |
| United Arab Emirates | 76 |
| Kazakhstan | 70 |
| Kuwait | 75 |
| Bahrain | 74 |
| Malaysia | 61 |
| Qatar | 65 |
| Burkina Faso | 63 |
| Lebanon | 61 |
| Chad | 52 |
| Bosnia and Herzegovina | 51 |
| Nigeria | 50 |

Source: worldpopulationreview.com.

with their neighbourhood of residence[4], in the year in which the final exam is taken, we use the dataset called *GBAADRESOBJECTBUS*. This dataset provides two codes (*soortobjectnummer* and *rinobjectnummer*) that uniquely identify the neighbourhood of residence of each individual who is registered in the Dutch population registration system. More specifically, we need to match these two codes with an additional code that identifies a neighbourhood (the variable *BU2021*, available in the dataset called *VSLGWTAB*). This neighbourhood code can be then used to obtain the geospatial data of the neighbourhood (from the *gemeentewijkbuurt* dataset)

[4]Spatial data at neighbourhood level (*buurt* in Dutch) represent the smallest geographical unit we could obtain from CBS.

or its socio-demographic characteristics (from the *wijk_en_buurtstatistieken* dataset).

We obtained the mosques' geolocation and characteristics from the website *moskeewijzer.nl* in November 2021[5]. Importantly, for each mosque, we know whether it is a Moroccan or Turkish mosque as reported by the website. In order to use these data in our analysis, we asked CBS to encrypt the address of the mosques so that each could get a *soortobjectnummer* and *rinobjectnummer*. We then matched again these codes with the corresponding neighbourhood identifier (*BU2021*). Finally, we computed the distance between the centroids of the students neighbourhood of residence and the closest neighbourhood with a mosque. In doing so, we considered only Turkish mosques for students with a Turkish migration background and only Moroccan mosque for students with a Moroccan background.

## C.3 Construction of outcome variables

We use two different datasets on outcomes in secondary education: a registry of the exam takers and exam outcomes (*EXAMVOTAB*) and a registry of the exam outcomes at course level (*EXAMVOVAKTAB*).

We start with the exam outcomes at the general level. First, we drop exam takers for which the exam outcomes are missing, while the exam taker did not withdraw from the exam. We only keep the students that passed or failed the final exams or that withdrew from the final exam. We standardize the average grade from the first central exams, the average grade from the central exams including the first and second retake (if applicable and grade improved) and the average grade from the continuous school exam by track with mean zero and standard deviation one. We generate a dummy that takes value one if the student passed the exams (and graduated).

For the data at course level (*EXAMVOVAKTAB*) we consider only the exam takers that are included in our analysis of *EXAMVOTAB*. The dataset contains 39 different exam subjects. We manually classified them into 29 subjects. Those exams that we could not classify into one of these subjects are dropped. The final list of exam subjects included in our analysis is the following: Arabic, Arts, Biology, Chemistry, Dutch, Economics, English, French, Frisian, Geography, German, Greek, History, Latin, Management, Math (VMBO level, A, B, C), Music, Philosophy, Physics, Physics and Chemistry (I and II), Russian, Social Studies, Spanish,

[5]The dataset used in our analysis contains the existing mosques in the Netherlands in the year 2021. As we do not know when a mosque was built, we use all the existing mosques in 2021 to compute the students' distance to the closest mosque. This introduces some measurement error as the map of mosques location has most likely changed between 2021 and 2014 (the first year considered in our analysis).

Technical Design, Turkish. This means that some practical exams and uncommon exams (both mainly in the pre-vocational track) are dropped from the sample. We merge these data with the date and time the exams are held, as reported by the website *www.examenblad.nl*.

## C.4 SIM survey

The *Survey integratie minderheden* (SIM) is a survey conducted by the *Sociaal en Cultureel Planbureau* (SCP) in collaboration with Statistics Netherlands. The survey includes questions on education, labour, social life, cultural integration, religion, health and sports. The interviews were conducted face-to-face, by telephone and online in 2006, 2011, 2015 and 2020.[6] The sample of the survey consists of respondents of 15 years or older of the four important (first and second generation) immigration groups in the Netherlands: Turkey, Morocco, the (former) Dutch Antilles, and Suriname (and in 2015 also Poland and Somalia), and of a control group of people without a migration background. Sample selection consisted of two steps: municipalities were randomly selected, within these municipalities the sample size was determined by municipality size, and people were selected randomly from the different immigration groups in the municipality.

In 2006, the data collection took place from March 2006 to December 2006. The sample consisted of 11,520 people, of whom 2,162 had a Turkish migration background and 2,390 a Moroccan migration background. The response rate was 53% (1,032 Moroccans and 1,132 Turks). In 2011, the data collection took place from November 2010 to June 2011. The sample consisted of 14,178 people, of whom 2,544 had a Turkish migration background and 2,826 a Moroccan migration background. The response rate was 48% (1,395 Moroccans and 1,347 Turks). In 2015, the data collection took place from January 2015 to July 2015. The sample consisted of 15,028 people, of whom 1,858 had a Turkish migration background and 2,082 a Moroccan migration background. The response rate was 45% (920 Moroccans and 951 Turks). In 2020, the data collection took place from September 2020 to January 2021.[7] The sample consisted of 15,498 people, of whom 2,020 had a Turkish migration background and 2,114 a Moroccan migration background. The response rate was 35.4% for individuals with a Turkish migration background (696 respondents) and 27.5% for individuals with a Moroccan migration

[6] The fieldwork period of each wave is given below. Due to measures against COVID-19 during Ramadan in 2020, mosques were closed and Muslims did not share the iftar meal with friends and neighbours.

[7] The data collection was delayed by the Covid-19 pandemic and took longer than planned because of lockdowns.

background (570 respondents).

We linked the SIM data to the administrative data from Statistics Netherlands. Some respondents are included in more than one survey wave and for those respondents we only keep the first observation. We are left with 5,400 respondents in total of a (first or second) generation Moroccan or Turkish migration background. In total, we have 2,670 respondents with a Moroccan migration background and 2,730 respondents with a Turkish migration background.

## C.5 Additional regressions

In this section we provide details for the regressions that we estimate in our heterogeneity analyses.

In section 6.2, we replicate our main results, using data at the subject level. To do so, we estimate the following regression:

$$\begin{aligned} y_{i,t,z} = {} & \alpha_{p,z} + \theta_{i,z} + \eta_{i,z} + \beta Treated_i * Ramadan\ Year_t + \zeta Treated_i \\ & + Exam\ Num_{i,t,z} + \delta X_{i,t} + \varepsilon_{i,t,z} \end{aligned} \tag{C1}$$

$y_{i,t,z}$ is the grade obtained by student $i$, in year $t$, in subject $z$, standardized at the subject-track-year level. $Exam\ Num_{i,t,z}$ represents fixed effects for the number of exams that student $i$ in year $t$ has already completed while taking exam $z$. $\alpha_{p,z}$ are school-track-exam subject fixed effects. $\theta_{i,z}$ are treatment type-exam subject-afternoon- two day exam fixed effects. The controls in the equation are the same as in the previous specifications. Standard errors are clustered at the school-track-subject-year level.

In section 7 we estimate the impact of Ramadan on exam performance, based on the timing of the exams and how many exams a student takes in a day. To do so, we employ the following regression:

$$\begin{aligned} y_{i,t,z} = {} & \alpha_{p,z} + \theta_{i,z} + \delta X_{i,t} + \gamma_t + Exam\ Num_{i,t,z} + \beta_1 DiD_{i,t} + \beta_2 Afternoon_z + \beta_3 Two\ Exam_z \\ & + \beta_4 DiD_{i,t} * Afternoon_z + \beta_5 DiD_{i,t} * Two\ Exam_{i,z} + \beta_6 Afternoon_z * Two\ Exam_{i,z} \\ & + \beta_7 DiD_{i,t} * Two\ Exam_z * Afternoon_z + \zeta Treated_i + \varepsilon_{i,t,z} \end{aligned} \tag{C2}$$

$y_{i,t,z}$ is the grade obtained by student $i$, in year $t$, in subject $z$, standardized at the subject-track-year level. $DiD_{it}$ is equal to the interaction between $Treated_i$ and $Ramadan\ Year_t$. $Afternoon_z$ is a dummy equal one if exam $z$ is in the afternoon. $Two\ Exam_{i,z}$ is a dummy

equal one if individual $i$ is taking exam $z$ on the same day as another exam. $Treated_i$ is replaced by individual fixed effects in some specifications. All the other variables are the same as in equation (C1).

In section 7 we estimate the impact of Ramadan on exam performance, depending on the week of Ramadan in which the exam is held. We estimate the following regression:

$$y_{i,t,z} = \alpha_{p,s,z} + \theta_{i,z} + \omega_{i,z} + \delta X_{i,t} + \gamma_t + Exam\ Num_{i,t,z} + \sum_{t=2014}^{2017} \beta_t Treated_i * Year_t + \zeta Treated_i$$
$$+ \sum_{n=0}^{3} Week_{z,n} + \phi_n Week_{z,n} * Treated_i + \varepsilon_{i,t,z} \quad \text{(C3)}$$

$y_{i,t,z}$ is the grade obtained by student $i$, in year $t$, in subject $z$, standardized at the subject-track-year level. $Week_{z,n}$ is a dummy taking value one if exam $z$ is held in week $n$ of the Ramadan month. $Week_{z,0}$ is equal to one if exam $z$ is held in 2018 in the week before the beginning of the Ramadan month. The coefficients of interests are $\phi_1$, $\phi_2$, $\phi_3$, which capture the impact of Ramadan exposure during the first, second or third week of the Ramadan month.

To test the possibility that Ramadan exposure affects students differently, depending on whether the exam is taken at the beginning or at the end of the Ramadan month, we also implement the following regression:

$$y_{i,p,s,t} = \alpha_{p,s} + \beta_1 Treated_i * Ramadan\ Year_t + \beta_2 Ramadan\ Days * Treated_i * Ramadan\ Year_t$$
$$+ \delta X_{i,t} + \gamma_t + \varepsilon_{p,s,t} \quad \text{(C4)}$$

In the equation above *Ramadan Days* is a variable measuring how many days after the beginning of Ramadan an exam is taken. All other variables are the same as in equation (1) in the main text. The coefficient $\beta_2$ measures the marginal effect of taking exams after an additional day of Ramadan exposure.
